# Supplementary material for: Site-specific anisotropic assembly of amorphous mesoporous subunits on crystalline metal–organic framework
Source: Nat Commun. 2023 Mar 3;14:1211. doi: 10.1038/s41467-023-36832-2 (PMC9984484; doi:10.1038/s41467-023-36832-2)
Supplement: Supplementary file 1 — Supplementary information [file 41467_2023_36832_MOESM1_ESM.pdf]

Supplementary Information

Site-specific anisotropic assembly of amorphous mesoporous subunits on crystalline metal–organic framework

Liu et al

Table of Contents

Supplementary Methods

Supplementary Figures1-33

Supplementary Tables 1-2

Supplementary References

Supplementary Methods

Synthesis of MIL-125 p-ANHs. The cake-like MIL-125 nanoparticles were prepared by using the hydrothermal method as described previously[1]. The MIL-125 p-ANHs were synthesized through the selective occupation strategy. In a typical process, 5.0 mg of MIL-125 nanoparticles were dispersed in 4.0 mL of ethanol, 6.0 mL of H_2O. Then, 200.0 mg of F-127 and 200.0 mg of dopamine hydrochloride were added and stirred to form a yellow solution. After that, 1.0 mL of TMB was added. The mixture was stirred for 30 min to form a stable emulsion, and then 0.5 mL of Tris aqueous solution (0.15 mM) was added. The reaction solution was left to react at room temperature for 12 hours. Then, the MIL-125 p-ANHs were centrifuged and washed with ethanol for three times. The final products were dispersed in 5.0 mL of ethanol.

Synthesis of mSiO_2&ZIF-8 p-ANHs. In a typical synthesis of mSiO_2&ZIF-8 p-ANHs, 15.0 mg of TRD ZIF-8, 80.0 mg of CTAB, 25 μL of triethanolamine, 30 mL of H_2O were added in a 50 mL three-neck flask. The mixture was stirred for 5 mins, then transferred into an oil bath at 60 °C. Then a mixture of 150 μL of TEOS and 7.0 mL of cyclohexane were added carefully on top of the solution to form a biphase system. The reaction was allowed to proceed for 12 h, then centrifuged, washed with water and ethanol. The final products were dispersed in 5.0 mL of ethanol.

Synthesis of PVP-coated CuO_2 nanodots. CuO_2 nanodots were synthesized through the one step process reported previously[2]. 500 mg of PVP was dissolved in 5.0 mL of $CuCl_2$ aqueous solution (0.01 M). Then, 5.0 mL of 0.02 M NaOH and 100 μL of H_2O_2 (30 wt.%) were added to the above mixture. After stirring for 30 min, the PVP coated CuO_2 nanodots were collected and washed with water for several times.

Loading CuO_2 nanodots and DSF into type 1 p-ANHs. 10.0 mL of type 1 p-ANHs aqueous dispersion (1 mg/mL) was mixed with 2.0 mL of CuO_2 aqueous solution (1 mg/mL) under stirring at 37 °C for 4 h, followed by centrifugation for 10 min. The resultant CuO_2-loaded type 1 p-ANHs were washed with water three times. Then, the obtained type 1-CuO_2 was dissolved in 10.0 mL of DSF ethanolic solution (5 mg/mL) and stirred for 12 h. The resulting CuO_2&DSF-loaded type 1 p-ANHs were collected by centrifugation (12,000 rpm, centrifugal force, 17726 g, 5 min) and washed three times with ethanol for further use.

In vitro cytotoxicity evaluation. The HeLa cells were treated with varying doses of DSF (0, 0.25, 0.5, 1, 2, and 4 μg/mL), CuO_2 nanodots (0, 0.25, 0.5, 1, 2, and 4 μg/mL), bare Type 1 p-ANHs (0, 3.125, 6.25, 12.5, 25, and 50 μg/mL) or CuO_2&DSF-loaded type 1 p-ANHs (0, 0.625, 1.25, 2.5, 5, and 10 μg/mL) for 24 h. The CCK-8 assay was carried out to determine the cell viability.

Synergistic chemo-photothermal therapy against HeLa tumor cells. The HeLa cells were divided into two groups for different treatments, including CuO_2&DSF-loaded type 1 p-ANHs and CuO_2&DSF-loaded type 1 p-ANHs + 808-nm laser. The HeLa cells were incubated with fresh medium containing different concentrations of CuO_2&DSF-loaded type 1 p-ANHs (0, 0.625, 1.25, 2.5, 5, and 10 μg/mL) for another 24 h. The power density of the laser irradiation was 1.5 W/cm^2. The CCK-8 viability assay was carried out to verify the synergistic chemo-photothermal effect.

Live-dead cell staining assay. The HeLa tumor cells were cultured in confocal dishes and exposed to various treatments, including control, DSF only, 808-nm laser only, CuO_2 only, CuO_2&DSF-loaded type 1 p-ANHs, and CuO_2&DSF-loaded type 1 p-ANHs + 808-nm laser. Laser exposure (1.5 W/cm^2) was implemented for 10 min. After washing with PBS (3×), the cells were incubated with calcein-AM (10 μL) and PI (5 μL) for 15 min. Finally, the cells were washed with PBS (3×) and visualized by CLSM.

Cell apoptosis analysis. The 4T1 cells were cultured in a six well plate for 24 h and exposed to various treatments, including control, DSF only, 808-nm laser only, CuO_2 only, CuO_2&DSF-loaded type 1 p-ANHs, and CuO_2&DSF-loaded type 1 p-ANHs. After washing, the cells were trypsinized, centrifuged, re-dispersed in PBS, and incubated with annexin V-FITC/PI for 20 min before conducting flow cytometry analysis.

Supplementary Figures

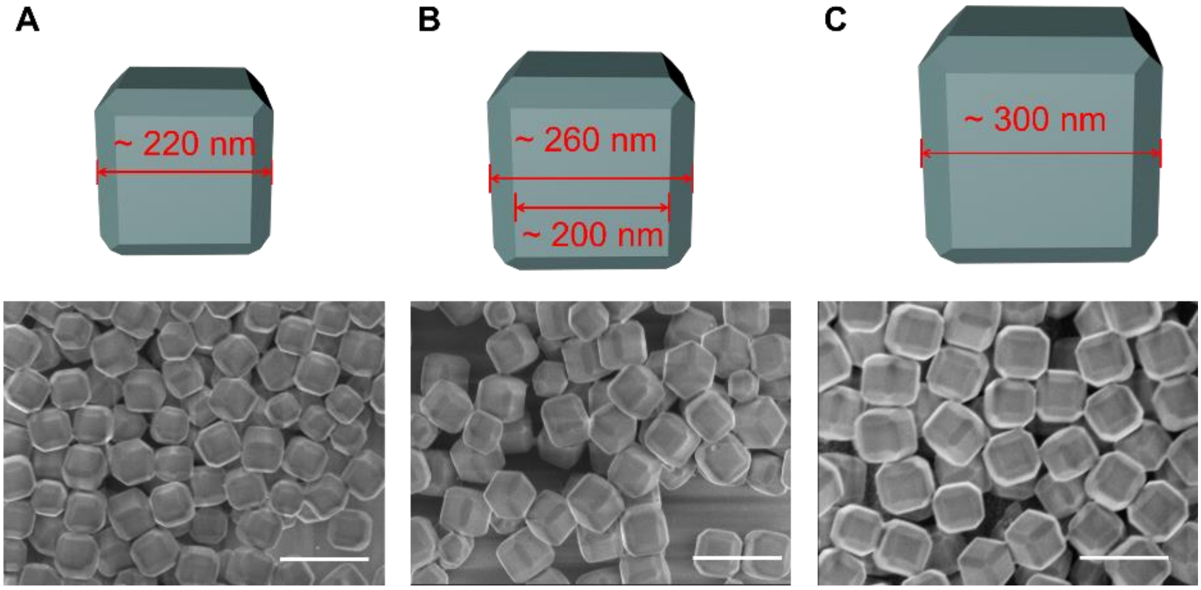

Supplementary Figure 1. The diagrams and SEM images of the TRD ZIF-8 nanoparticles with different diameters: (A) ~220 nm; (B) ~260 nm; (C) ~300 nm. Scale bars: 500 nm for (A-C). The diameters of the TRD ZIF-8 nanoparticles are defined as the average edge length of the corresponding equivalent cube.

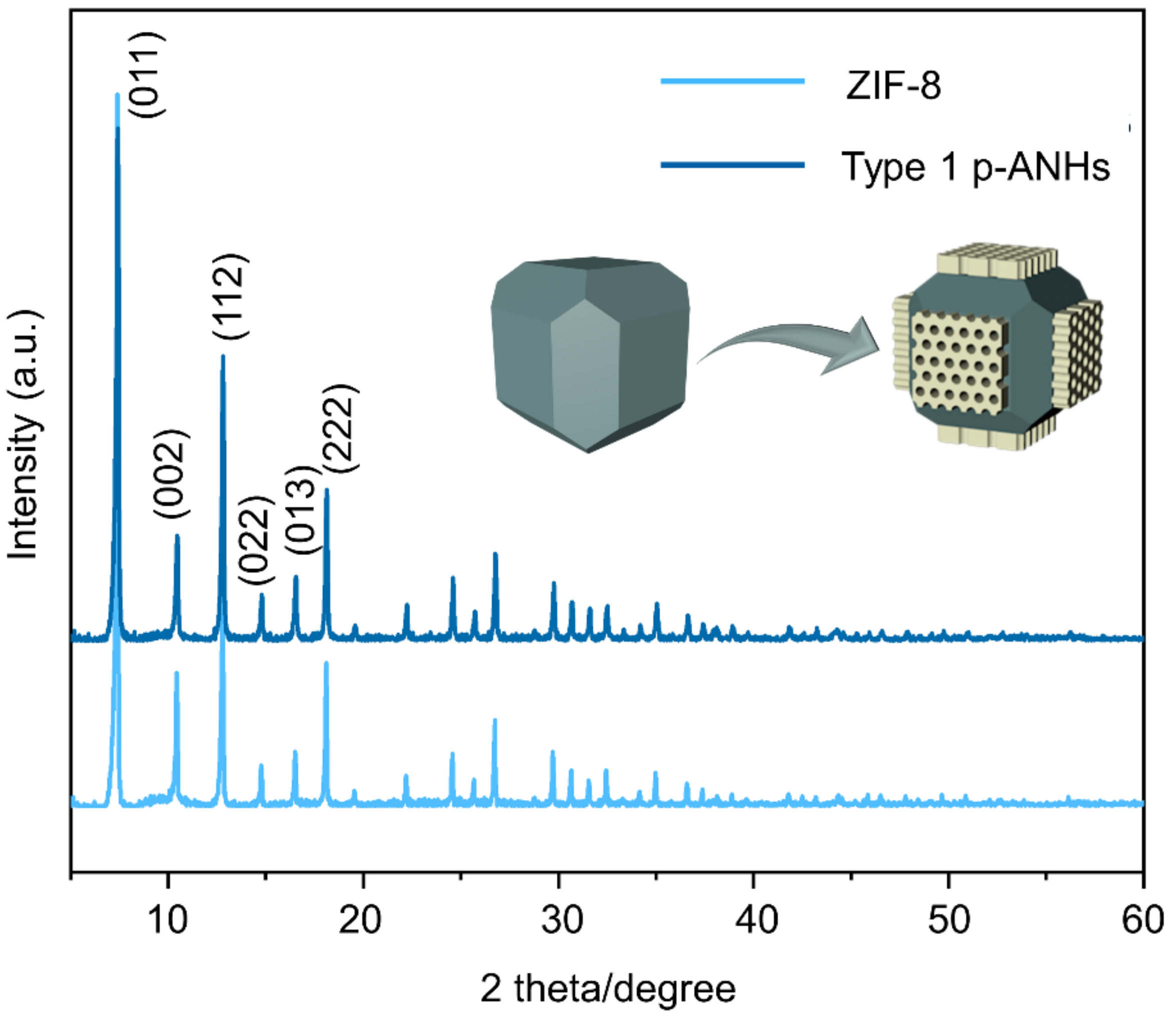

Supplementary Figure 2. XRD patterns of the pristine ZIF-8 host nanoparticles and the type 1 p-ANHs. The XRD pattern of the obtained type 1 p-ANHs shows the identical diffraction peaks as pristine ZIF-8 host, conforming the good crystallinity of ZIF-8 building blocks in the nanohybrids, and indicating that the selective growth of amorphous mPDA subunits do not affect the crystal structure of the ZIF-8 host. Source data are provided as a Source Data file.

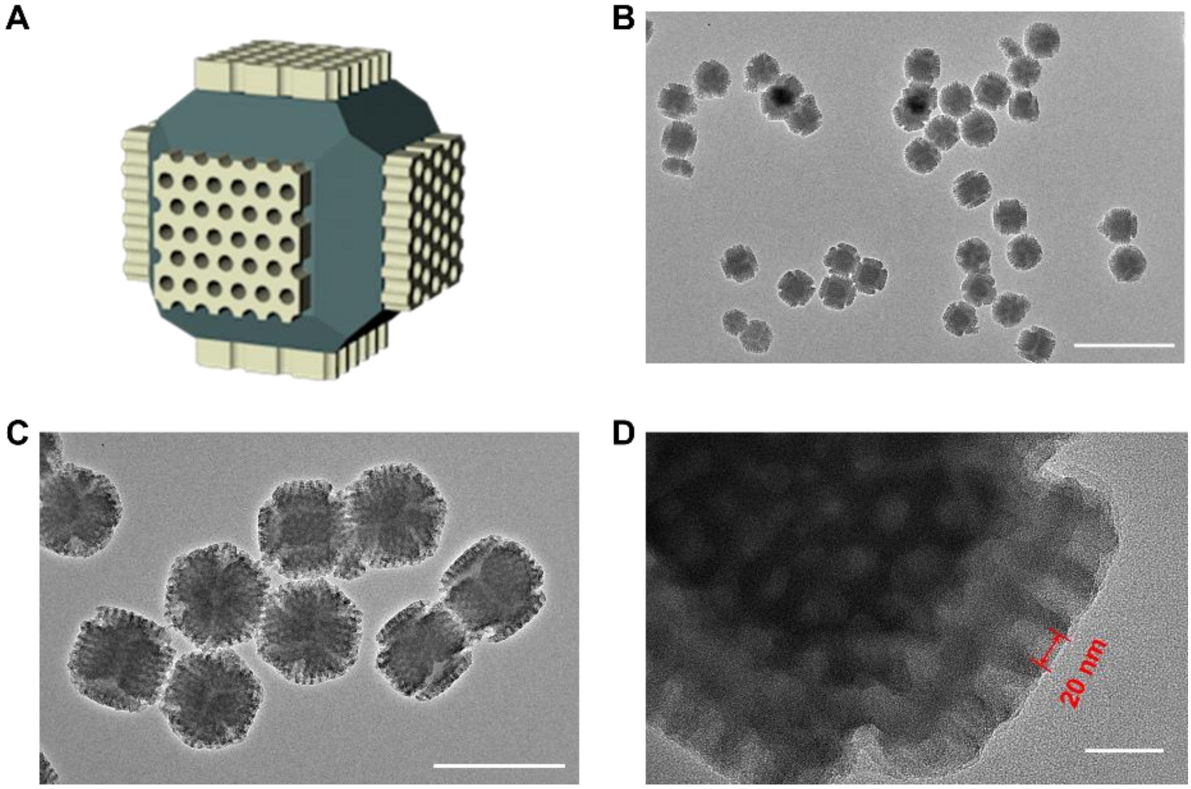

Supplementary Figure 3. (A) The diagrams and (B) TEM images with different magnifications of the type 1 p-ANHs. Scale bars: 1 μm for (B), 500 nm for (C), 50 nm for (D). The nanohybrids are very uniform and monodispersed, which are composed by dual-building blocks of mPDA nanoplates with vertical mesopore channels and TRD ZIF-8. The pore size of the mPDA nanoplates is about 20 nm, and the thickness of the nanoplates is about 40 nm.

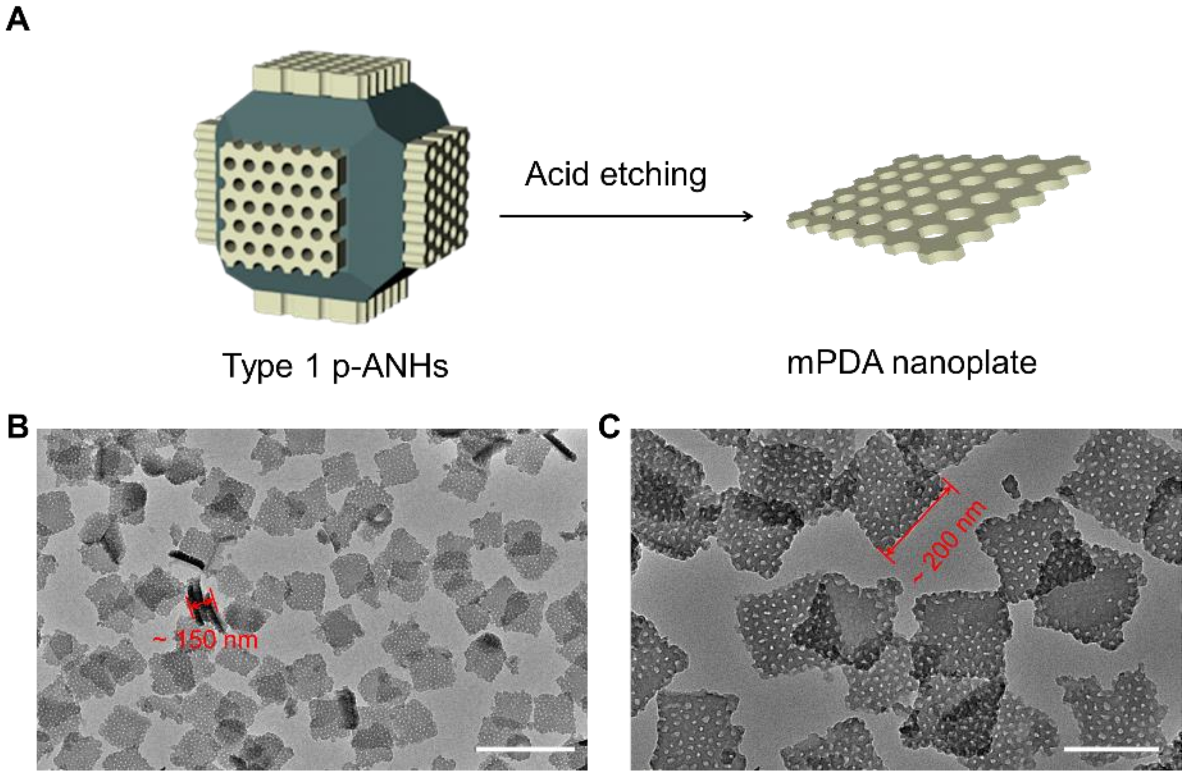

Supplementary Figure 4. (A) The schematic illustration for the synthesis of mPDA nanoplates by acid etching of ZIF-8 building blocks from type 1 p-ANHs. (B, C) TEM images with different magnifications of the obtained mPDA nanoplates. Scale bars: 500 nm for (B), 200 nm for (C). The uniform and well-dispersed square slices of mPDA are obtained after etching the MOF building blocks with acid, indicating that the mPDA subunits on the MOF host are isolated from each other without cross-linking. The thickness of the mPDA nanoplates is 40~50 nm. The width of the mPDA nanoplates is about 200 nm, consisting with the side length of the {100} facets of pristine TRD ZIF-8 host.

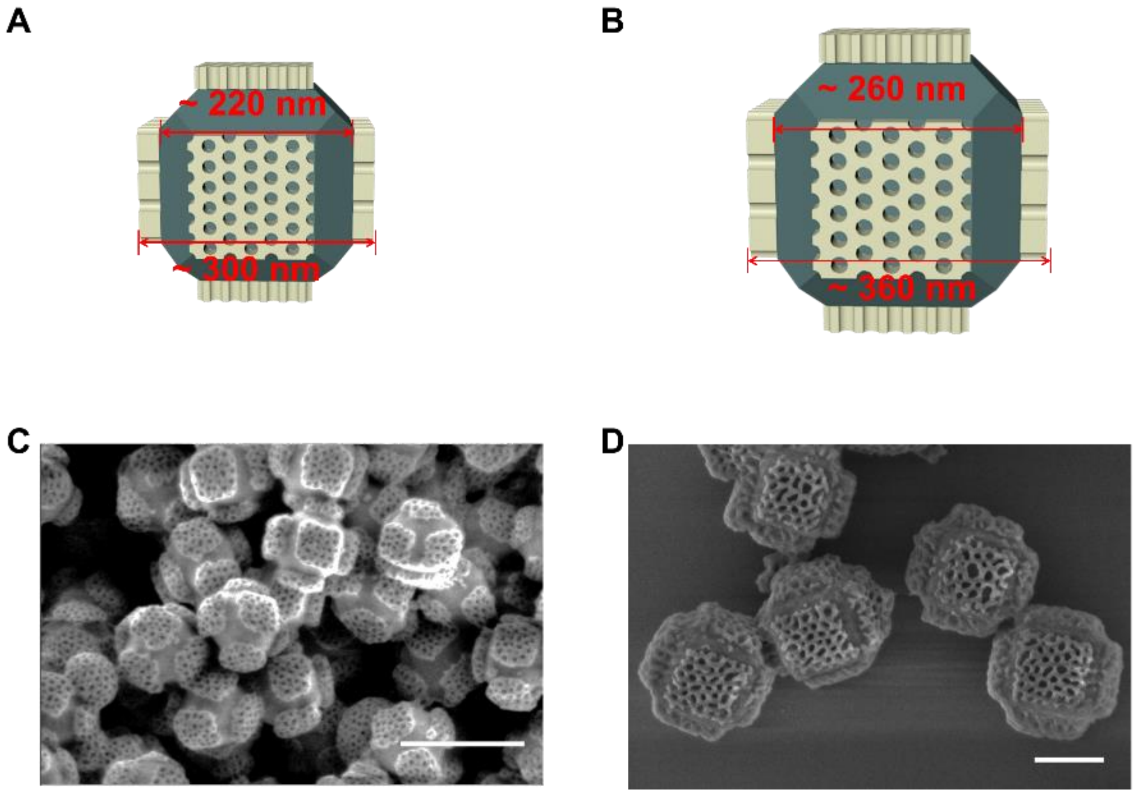

Supplementary Figure 5. (A, B) The diagrams and (C, D) SEM images of type 1 p-ANHs with different diameters. Scale bars: 500 nm for (C), 200 nm for (D). The diameter of the type 1 p-ANHs can be well tuned by controlling the size of the pristine ZIF-8 host. It can be seen that whether it is grown on large or small sized ZIF-8 host, the corresponding nanohybrids with type 1 architecture can be controllably synthesized, demonstrating the universality of the selective occupation strategy.

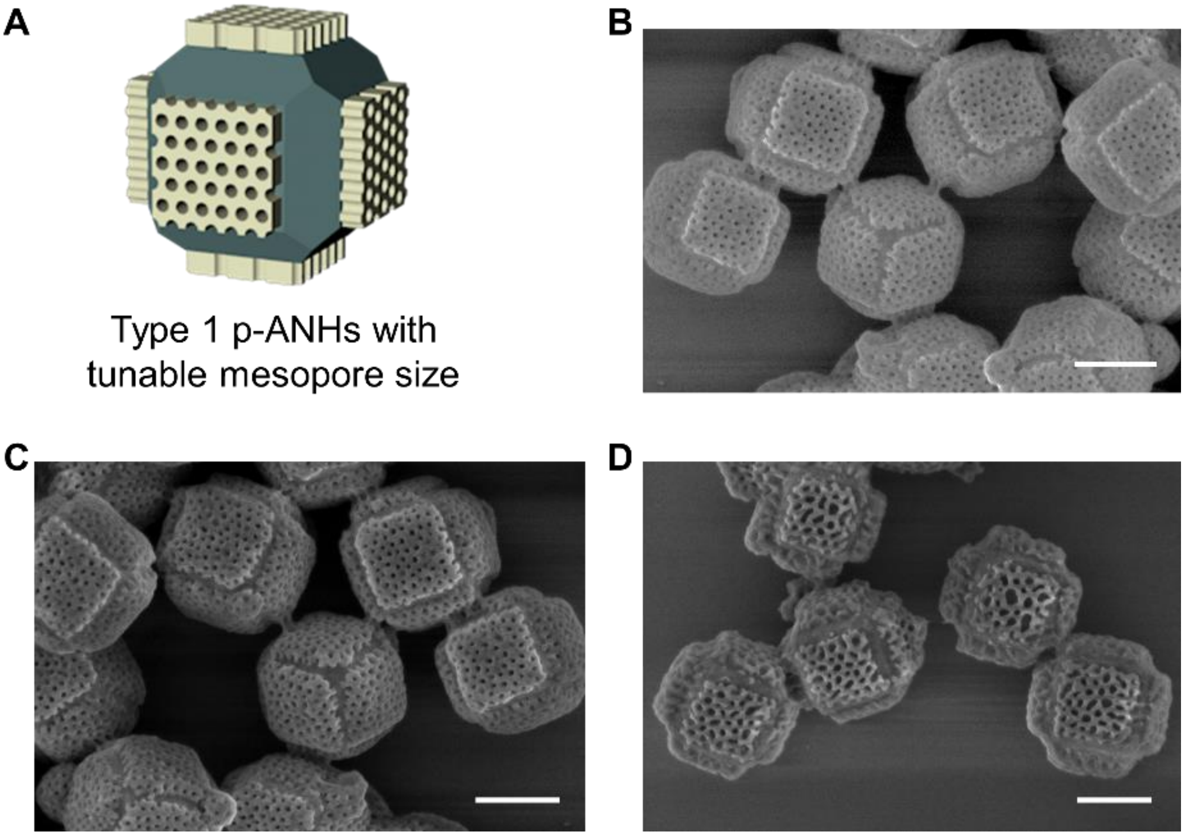

Supplementary Figure 6. (A) The diagram and (B-D) SEM images of type 1 p-ANHs with tunable mesopore sizes: (B) 5 nm; (C) 10 nm; (D) 20 nm. Scale bars: 200 nm for (B-D). The mesopore size of mPDA subunits can be tuned by varying the ratio of TMP/TMB in the oil phase. Without the addition of TMP, the mesopore size is about 5 nm. When the amount of TMP in the oil phase is increased to 150 and 200 mg, the pore size can be adjusted to 10 and 20 nm, respectively. It worth noting that the amount of TMP must be less than 200 mg for the synthesis of type 1 p-ANHs.

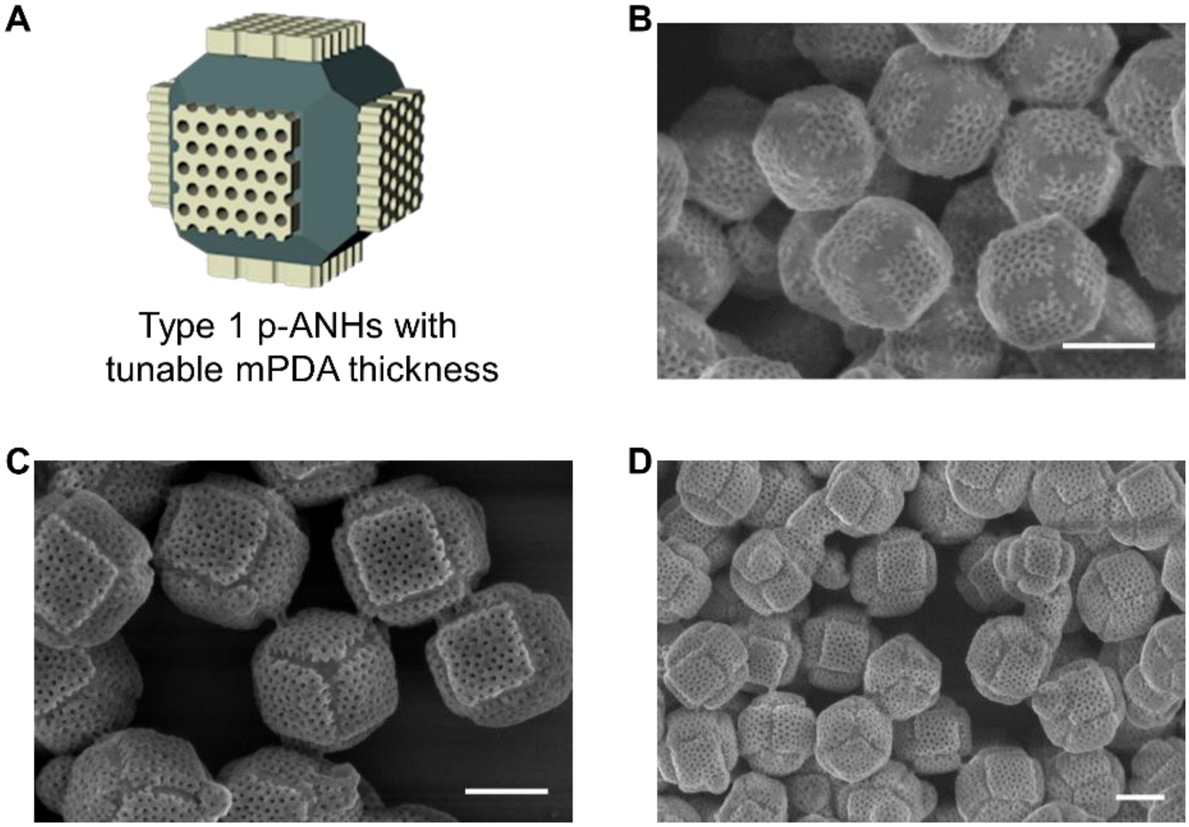

Supplementary Figure 7. (A) The diagram and (B-D) SEM images of type 1 p-ANHs with tunable mPDA subunits' thickness: (B) ~15 nm; (C) ~40 nm; (D) ~70 nm. Scale bars: 200 nm for (B-D). The thickness of mPDA subunits can be adjusted in the range from 15 to 70 nm by tuning the concentration of the dopamine precursor, which provides convenience for us to regulate the proportion of mesopores and micropores in the type 1 p-ANHs.

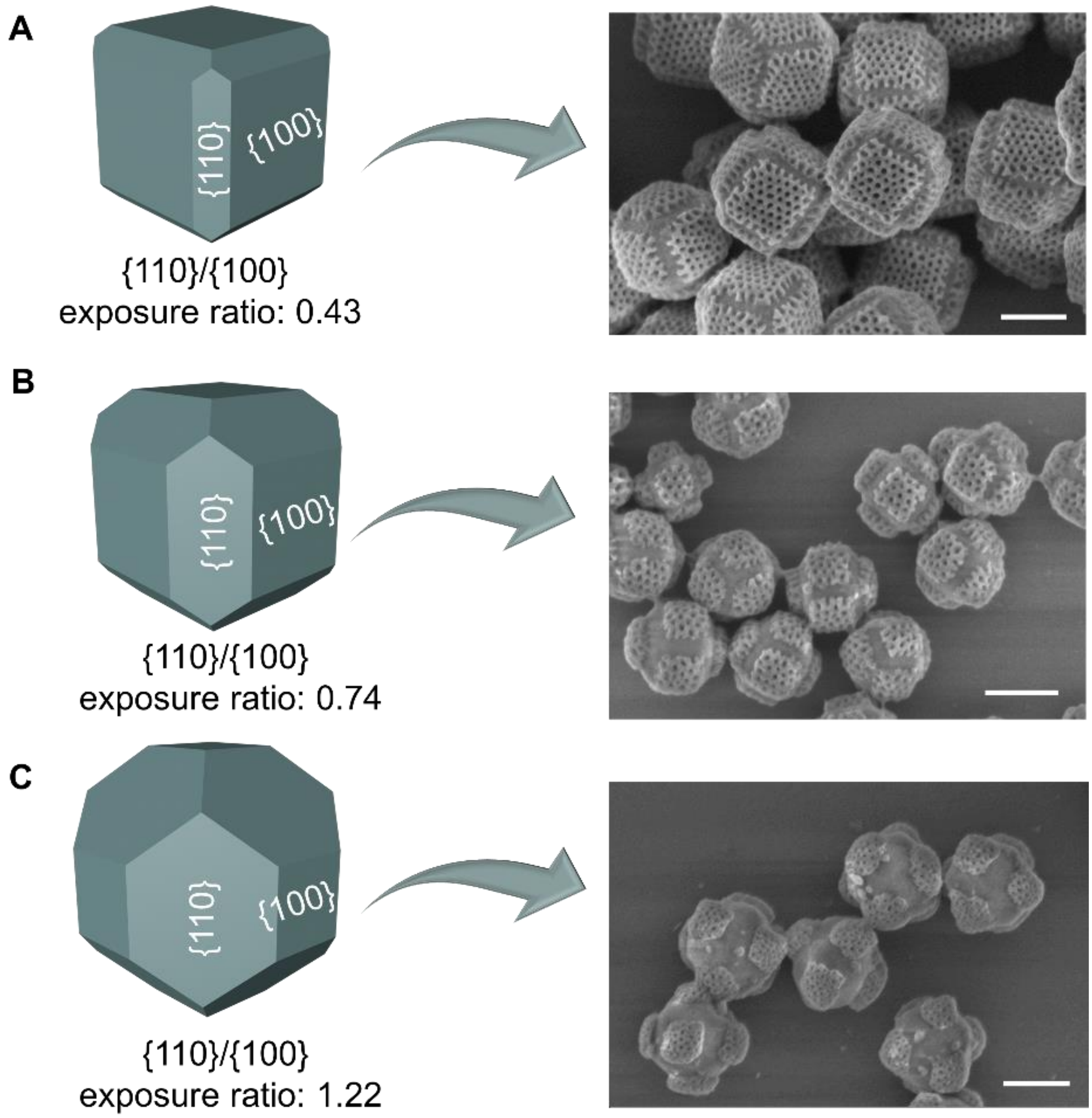

Supplementary Figure 8. The selective growth of mPDA nanoplates on the {100} facets of pristine TRD ZIF-8 host with different {110}/{100} exposure ratio: (A) 0.43; (B) 0.74; (C) 1.22. Scale bars: 200 nm for (A-C). Regardless of {110}/{100} exposure ratio, mPDA nanoplates can recognize {100} planes of the pristine ZIF-8 host and selectively grow on it, indicating the university of the selective occupation strategy. It provides another way for us to regulate the proportion of mesopores and micropores in the type 1 p-ANHs.

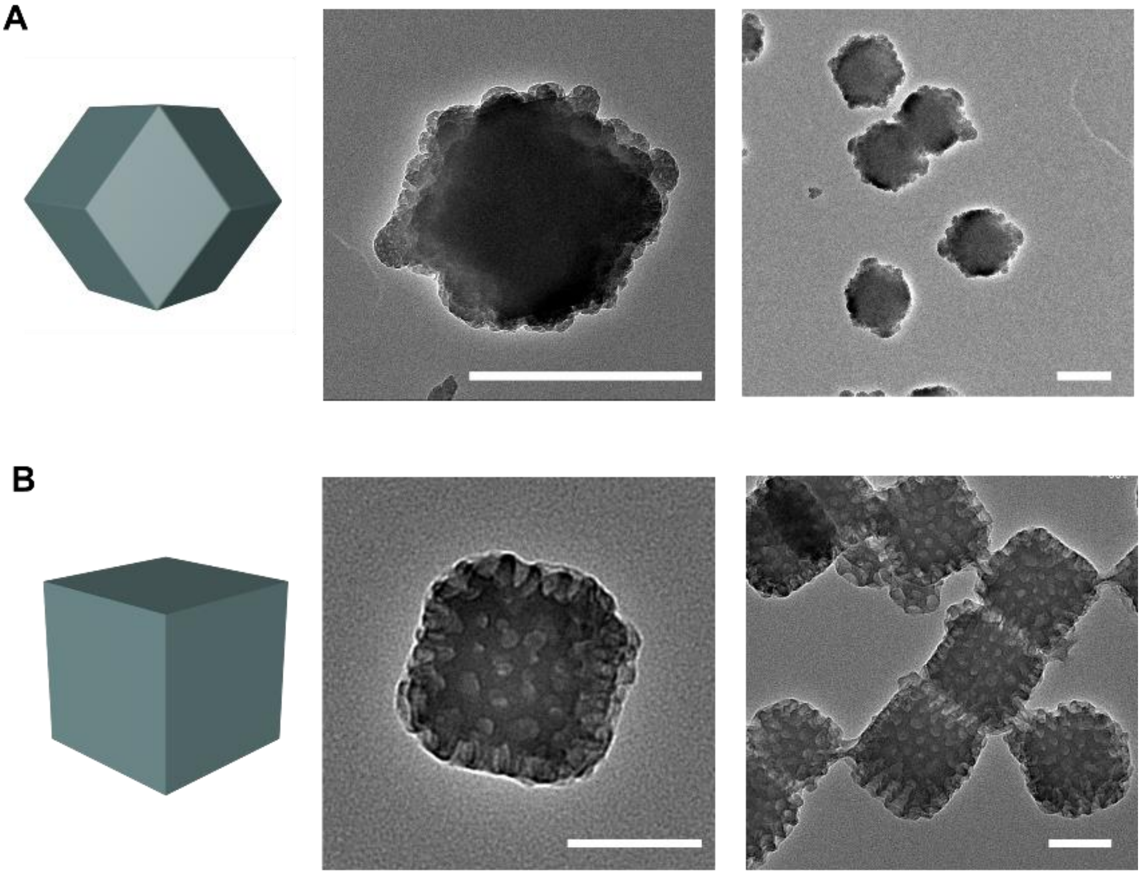

Supplementary Figure 9. The selective growth of mPDA nanoplates on the {100} facets of pristine TRD ZIF-8 host with different {100}/{110} exposure ratio: (A) ~0%; (B) ~100%. Scale bars: 500 nm for (A); 100 nm for (B). For type 1 growth, if RD ZIF-8 is used as host, the growth manner of mPDA is changed greatly. Instead of the layer growth to form the mPDA nanoplates, many mPDA nanoparticles varying sizes are deposited on the {110} facet of RD ZIF-8. If cubic ZIF-8 is used as host for the type 1 growth, mPDA nanoplates grow completely on the {100} facets of the nanocube to form the core@shell structure.

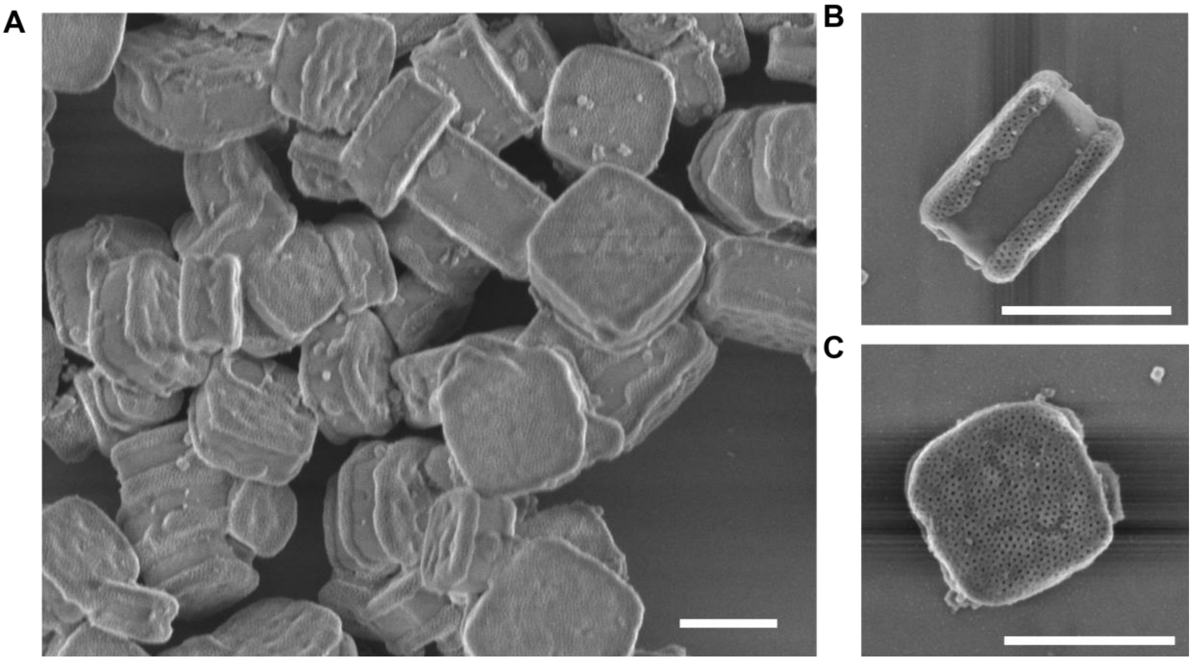

Supplementary Figure 10. (A) SEM image of MIL-125 based p-ANHs synthesized by the selective growth of mPDA on {001} facets of MIL-125. (B, C) SEM images of a single MIL-125 p-ANHs taken from different perspectives. Scale bars: 500 nm.

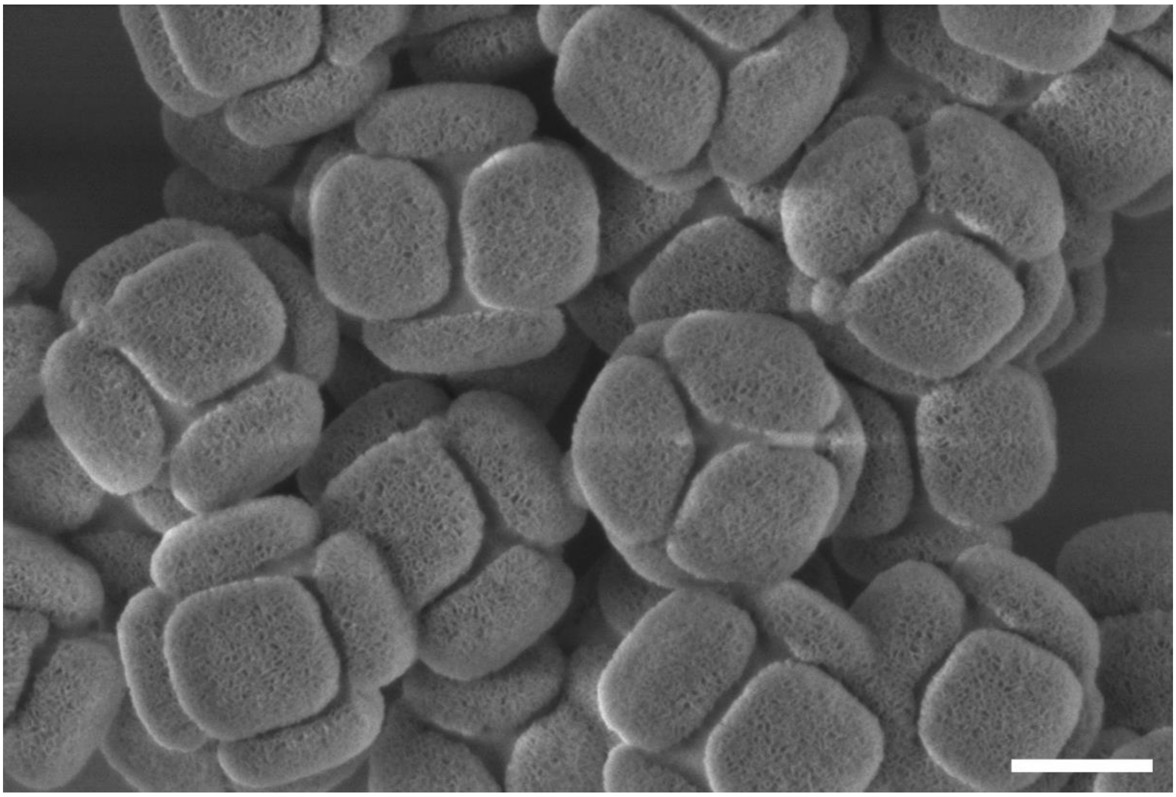

Supplementary Figure 11. SEM image of $mSiO_2$&ZIF-8 p-ANHs. It can be seen that amorphous $mSiO_2$ is selectively grown on {100} facets of TRD ZIF-8. Scale bars: 200 nm.

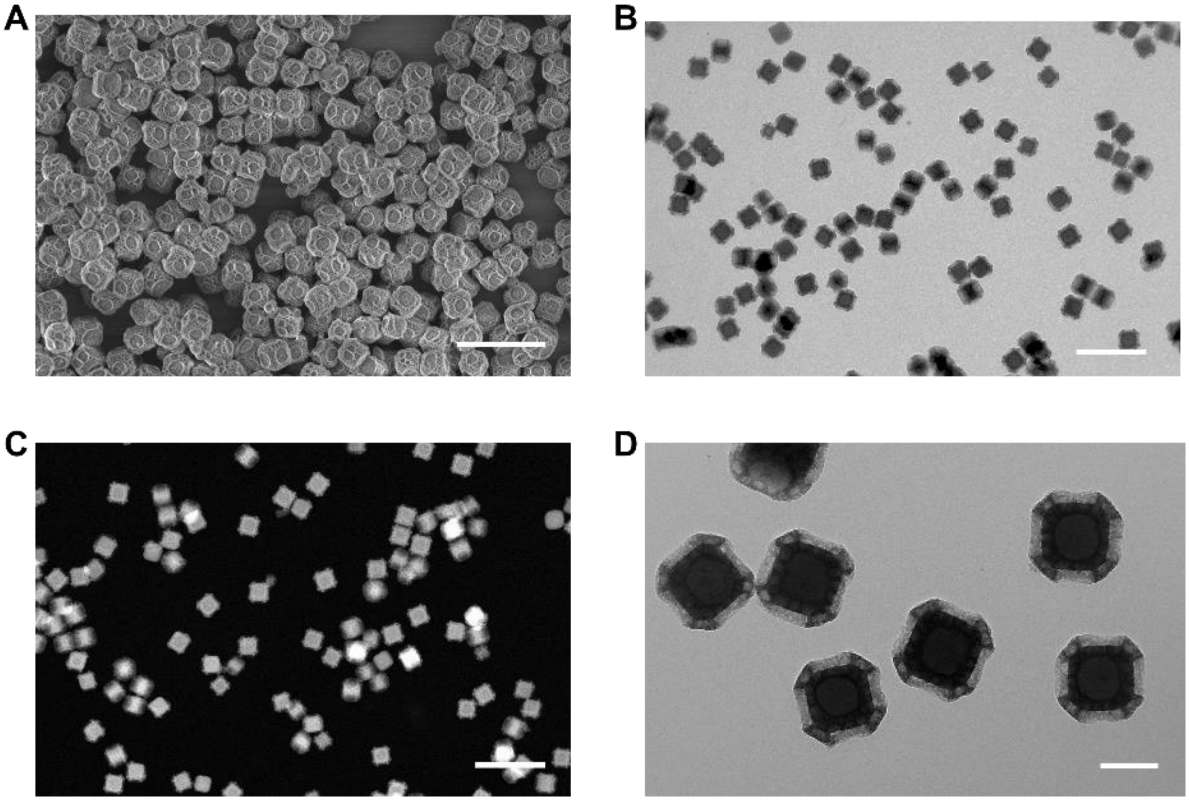

Supplementary Figure 12. (A) The SEM, (B, D) TEM and (C) HAADF-STEM images of the type 2 p-ANHs. Scale bars: 1 μm for (A-C); 200 nm for (D). It can be seen that the nanohybrids are uniform and well dispersed. type 2 p-ANHs are composed of cubical mPDA skeleton with vertical mesopore channels and TRD ZIF-8 building block in the cubical skeleton.

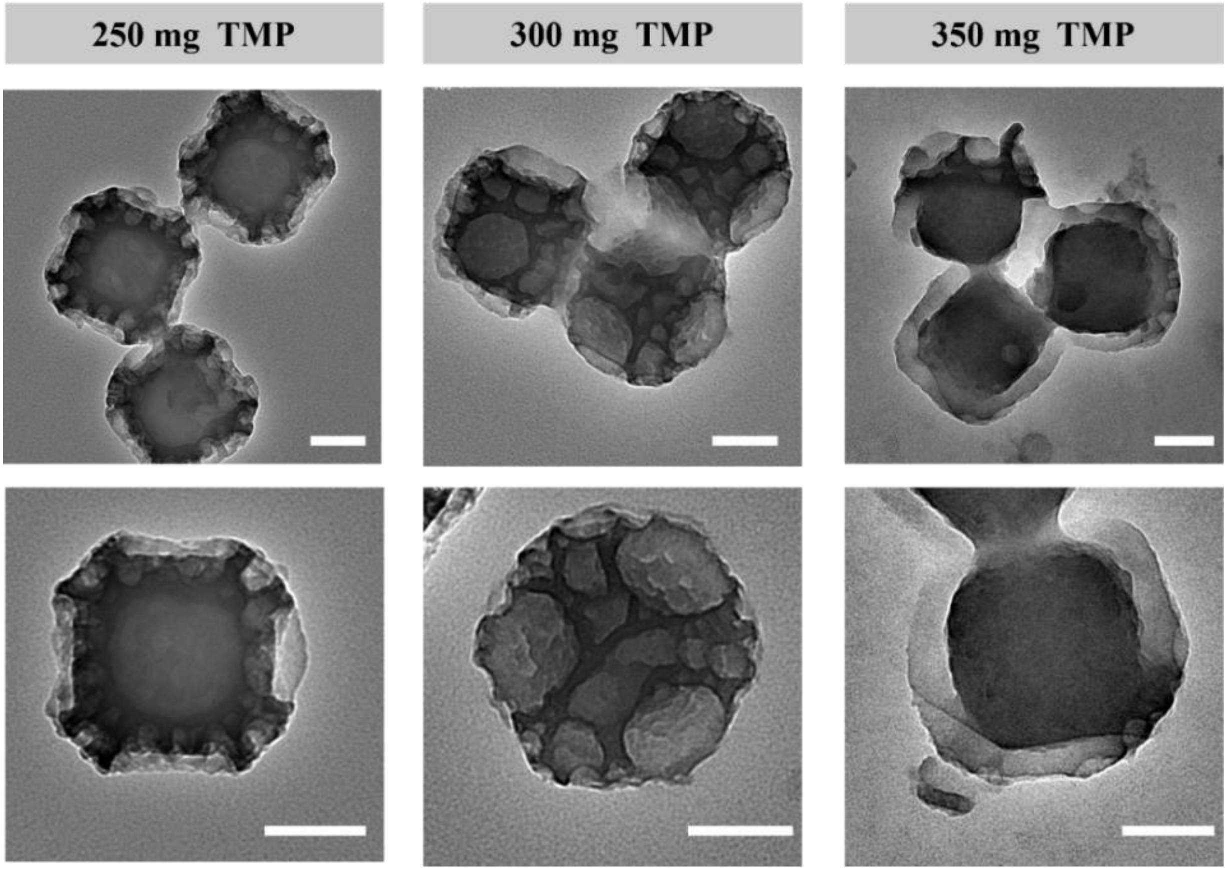

Supplementary Figure 13. TEM images of type 2 p-ANHs with tunable mesopore sizes. Scale bars: 100 nm. The mesopore size of mPDA subunits can be tuned by varying the ratio of TMP/TMB in the oil phase. As the amount of TMP in the oil phase increases from 200 to 300 mg, the pore size can be adjusted from 20 to 80 nm. It worth noting that the amount of TMP must be more than 200 mg for the synthesis of type 2 p-ANHs.

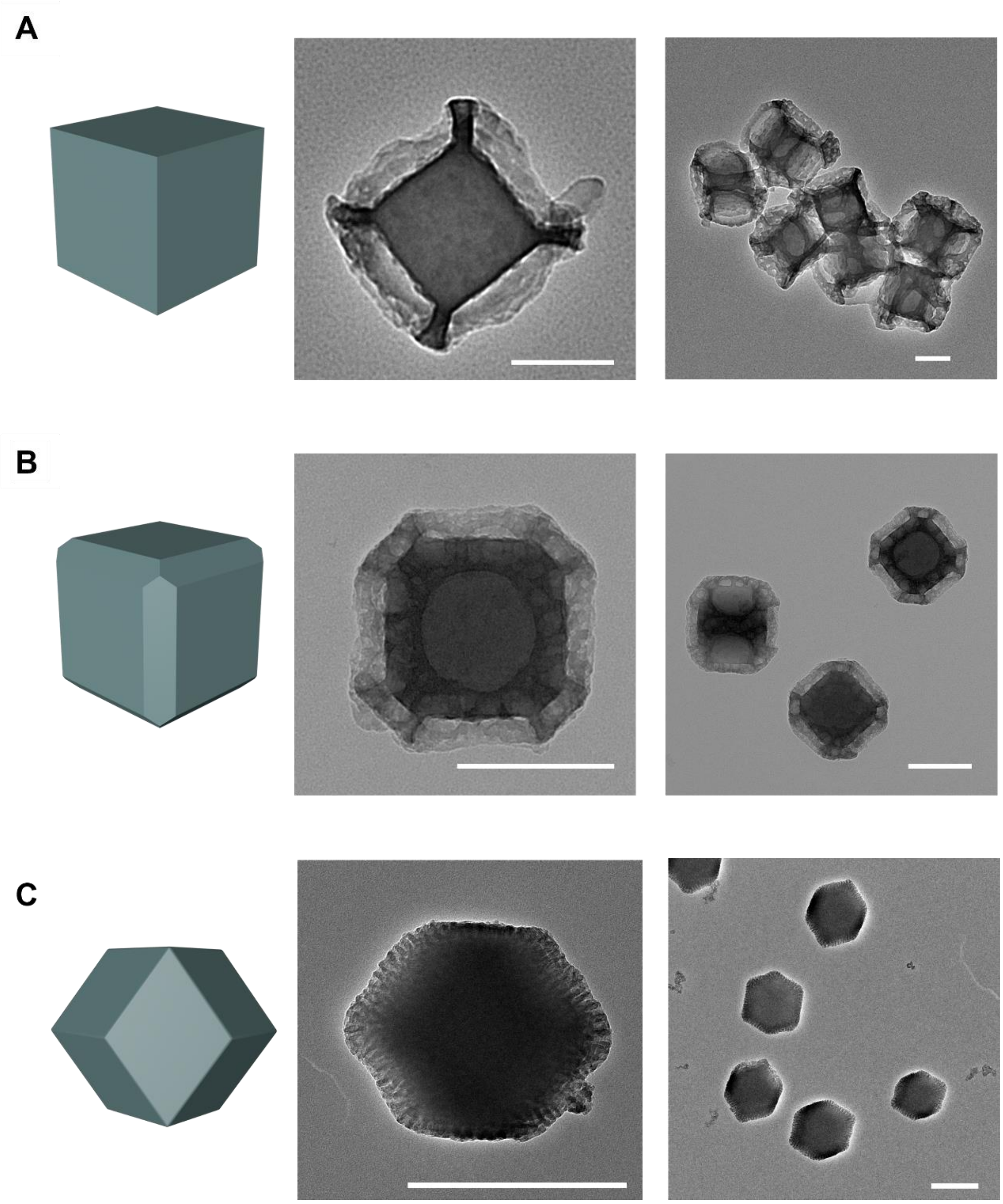

Supplementary Figure 14. The selective growth of mPDA nanoplates on the {110} facets of pristine TRD ZIF-8 host with different {110}/{100} exposure ratio: (A) ~0%; (B) ~43%; (C) ~100%. Scale bars: 200 nm for (A); 100 nm for (B); 500 nm for (C). When RD ZIF-8 with 100% {110} facets exposure is used as host, mPDA subunits grow completely on the {110} facets of RD ZIF-8 to form the core@shell structure. Regardless of {110}/{100} exposure ratio, even close to 0%, mPDA nanoplates can selectively grow on {110} planes of ZIF-8 host, indicating the university of the selective occupation strategy.

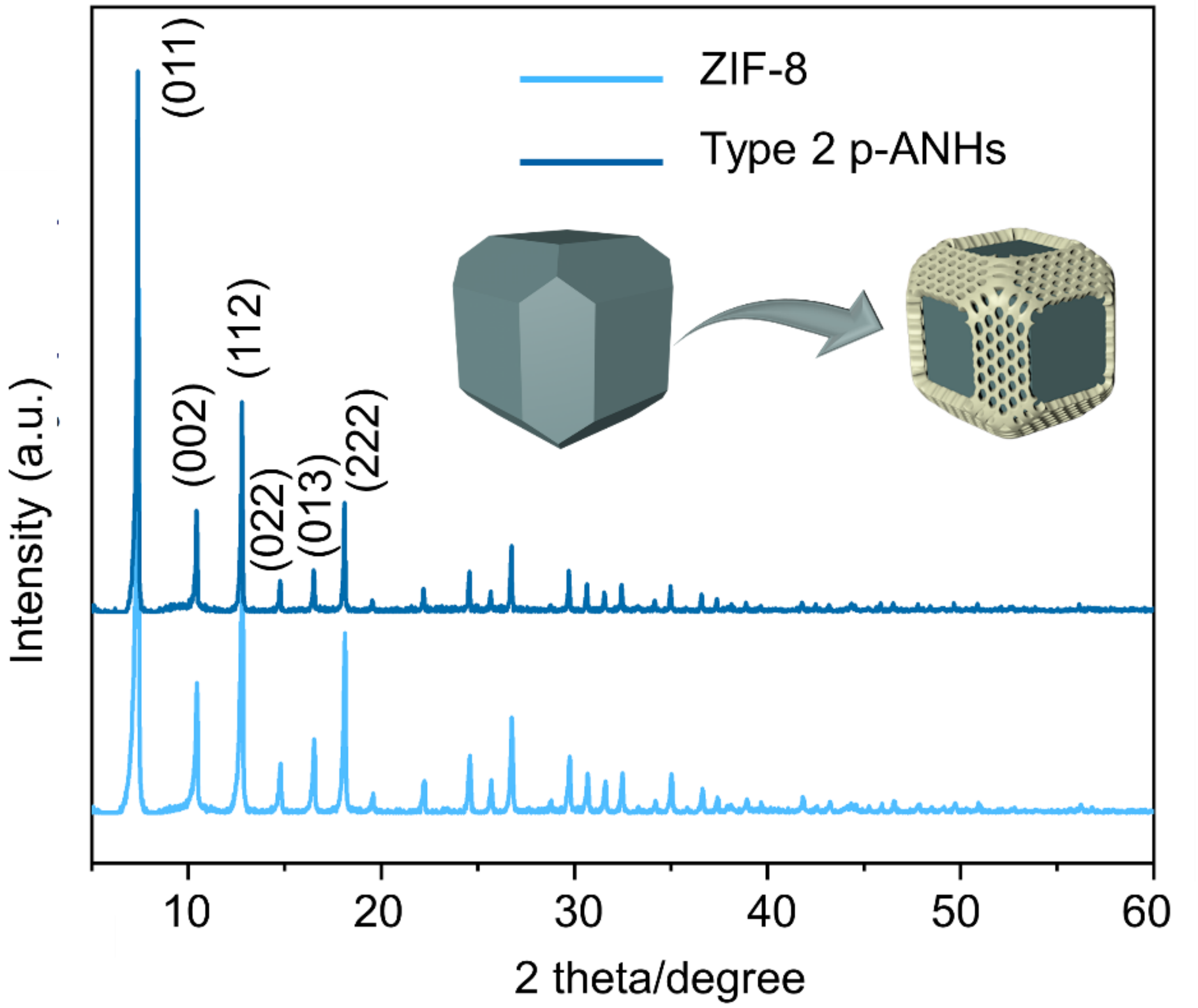

Supplementary Figure 15. (A) XRD patterns of the pristine ZIF-8 host nanoparticles and the type 2 p-ANHs. The XRD pattern of the obtained type 2 p-ANHs shows the identical diffraction peaks as pristine ZIF-8 nanoparticles, conforming the good crystallinity of ZIF-8 building blocks in the nanohybrids, and indicating that the selective growth of amorphous mPDA subunits do not affect the crystal structure of the ZIF-8 host. Source data are provided as a Source Data file.

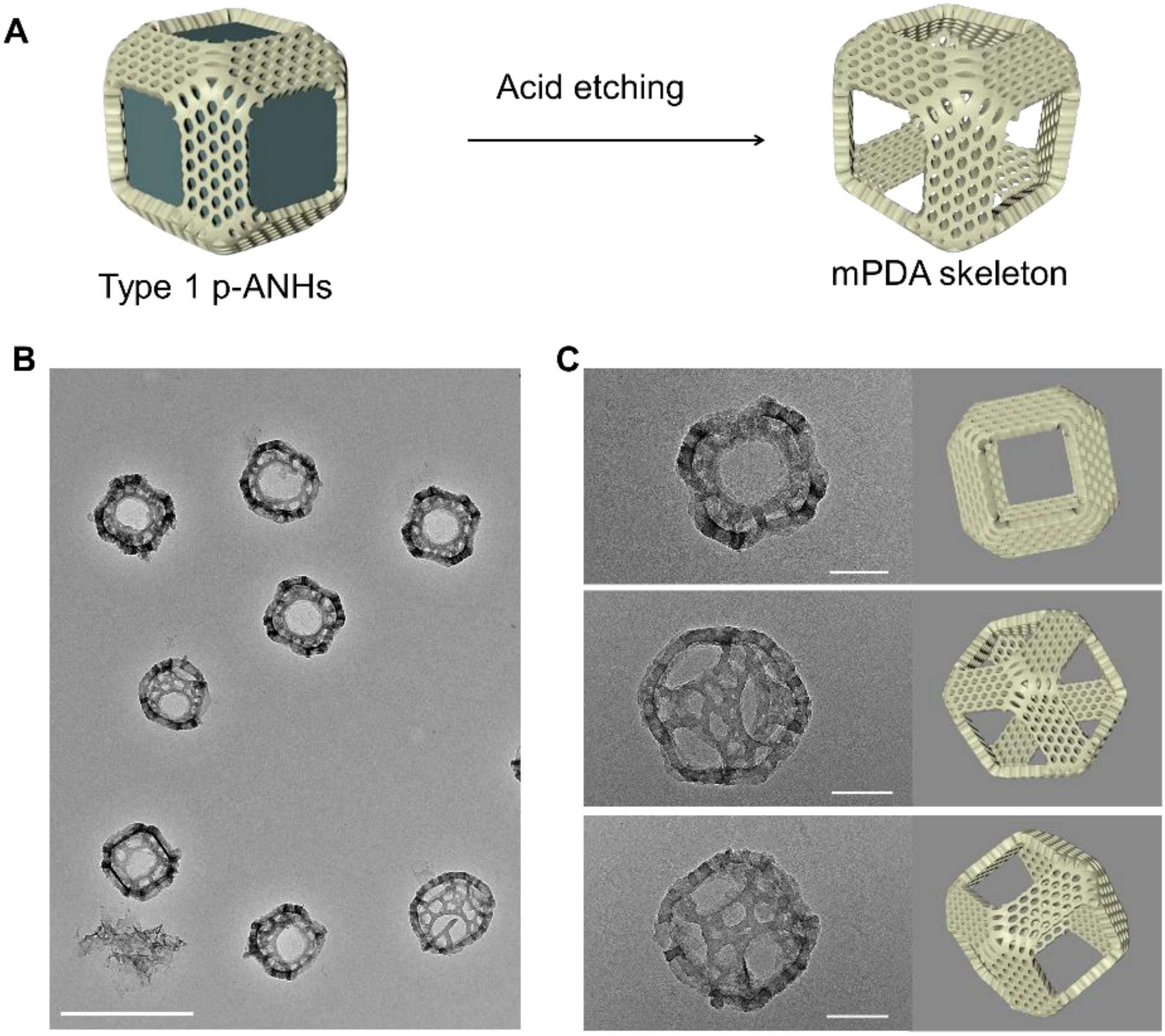

Supplementary Figure 16. (A) The schematic illustration for the synthesis of hollow mPDA cubical skeletons by acid etching of ZIF-8 building blocks from type 2 p-ANHs. (B-D) TEM images with different magnifications of the obtained hollow mPDA cubical skeletons. Scale bars: 500 nm for B, 100 nm for C. The hollow mPDA cubical skeleton can be well retained after etching the MOF building blocks with acid. The size of the mesopores on the skeletons is not uniform, in the range of 15 to 40 nm.

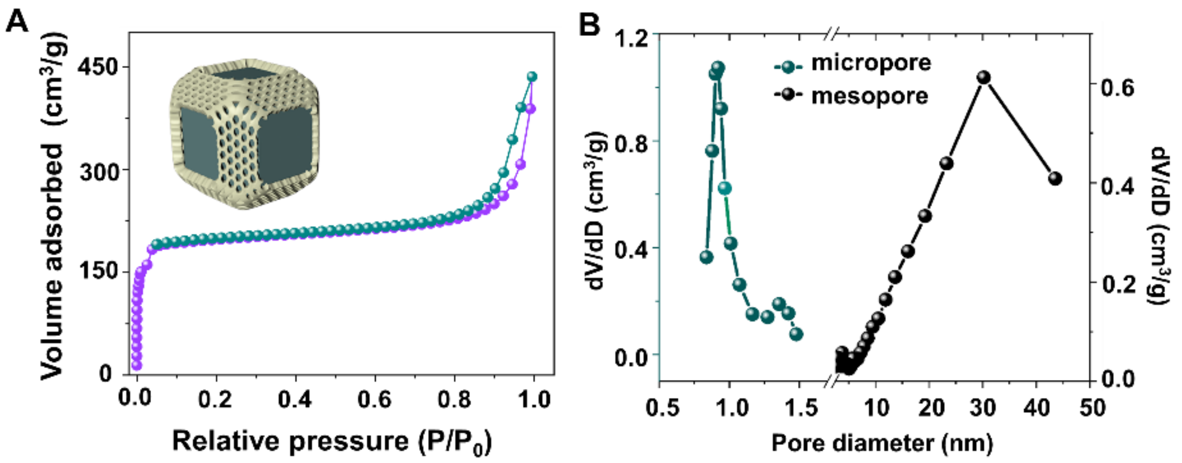

Supplementary Figure 17. (A) Nitrogen sorption isotherms and (B) corresponding pore size distribution of the type 2 p-ANHs. Nitrogen sorption isotherm shows the combination of type I and type IV isotherms with obvious nitrogen adsorption capacity at relative pressures of 0~0.05 and 0.70~0.95, indicating the presence of both micropores and mesopores in the nanohybrids. The pore size distribution curve shows a narrow peak at about 0.8 nm and a broad peak at 10~50 nm, which can be attributed to the micropores in the ZIF-8 building blocks and the mesopores in the mPDA nanoplates, respectively. Source data are provided as a Source Data file.

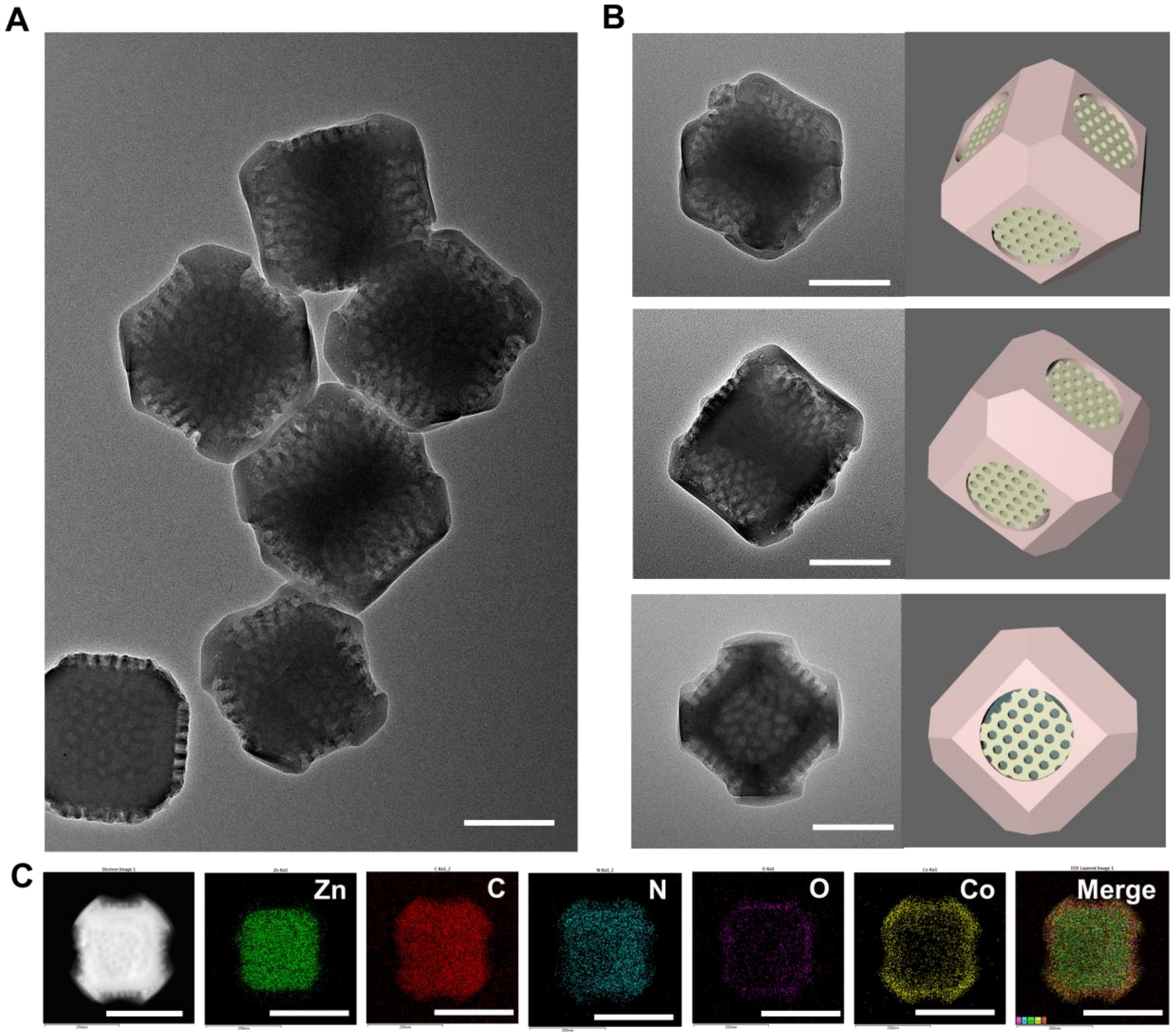

Supplementary Figure 18. (A) TEM image of type 3 p-ANHs. (B) TEM images and the corresponding diagrams of a single type 3 nanoparticle taken from different perspectives. (C) EDX elemental mapping of one typical type 3 p-AHN. Scale bars: 200 nm for (A) and (B), 250 nm for (C). Using type 1 p-ANHs as seeds, the tertiary building blocks of ZIF-67 (Co^{2+}) can be epitaxially grown on the exposed {110} facets by directly immersing the seeds into the 2-methylimidazole and Co^{2+} ions methanol solution. The obtained type 3 p-ANHs display a TRD morphology, and the nanoparticles are uniform and well dispersed. Different from the mPDA patches in type 1 p-ANHs seeds, the mPDA nanoplates are partially covered by the tertiary building blocks of ZIF-67 to form the inlay structure. The Co element is mainly distributed around the nanoparticles, which is consistent with the structural model.

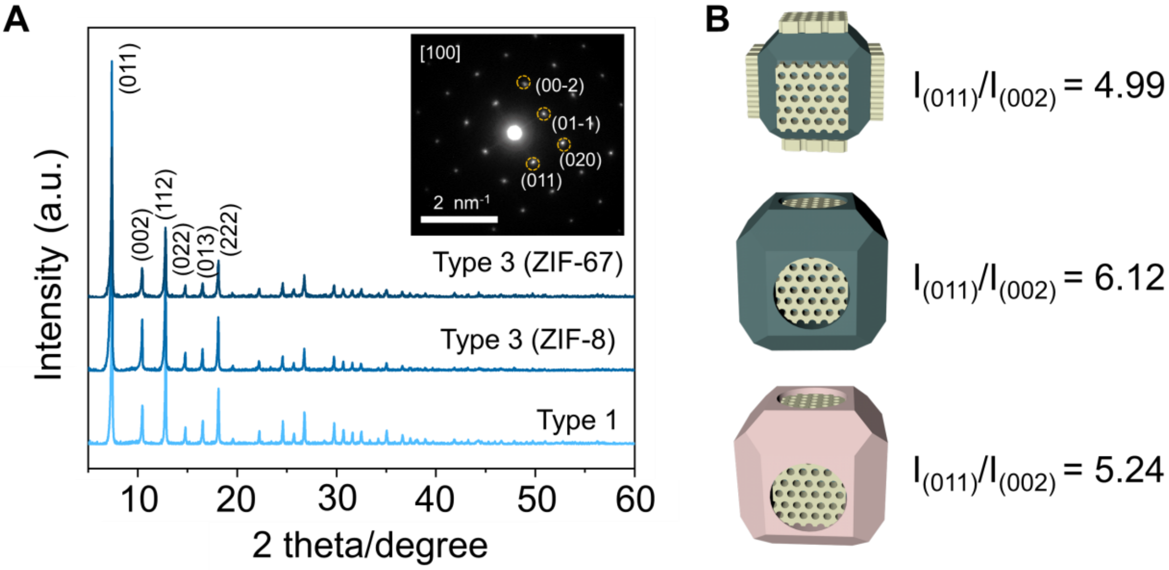

Supplementary Figure 19. XRD patterns of the type 3 p-ANHs after the epitaxial growth of tertiary ZIF-8 or ZIF-67 building block on the {110} facets of type 1 p-ANHs. Because of the identical crystal structure of ZIF-8 and ZIF-67, type 3 p-ANHs with different composites exhibit the similar diffraction peaks. The peak intensity ratio between {011} and {002} facets of type 3 and 4 p-ANHs are about 6.12 and 5.24, respectively, which are larger than that of the type 1 p-ANHs seeds (4.99). The increased peak intensity ratio between {011} and {002} facets can be attributed to the oriented growth of tertiary ZIF-8 or ZIF-67 along the <011> direction, which are consistent with our rational design. The inset in A is the SAED patterns taken from an entire type 3 p-AHN along [100] zone axis, indicating the single single-crystalline nature of the obtained type 3 p-ANHs and the epitaxial growth manner of the tertiary building blocks. Source data are provided as a Source Data file.

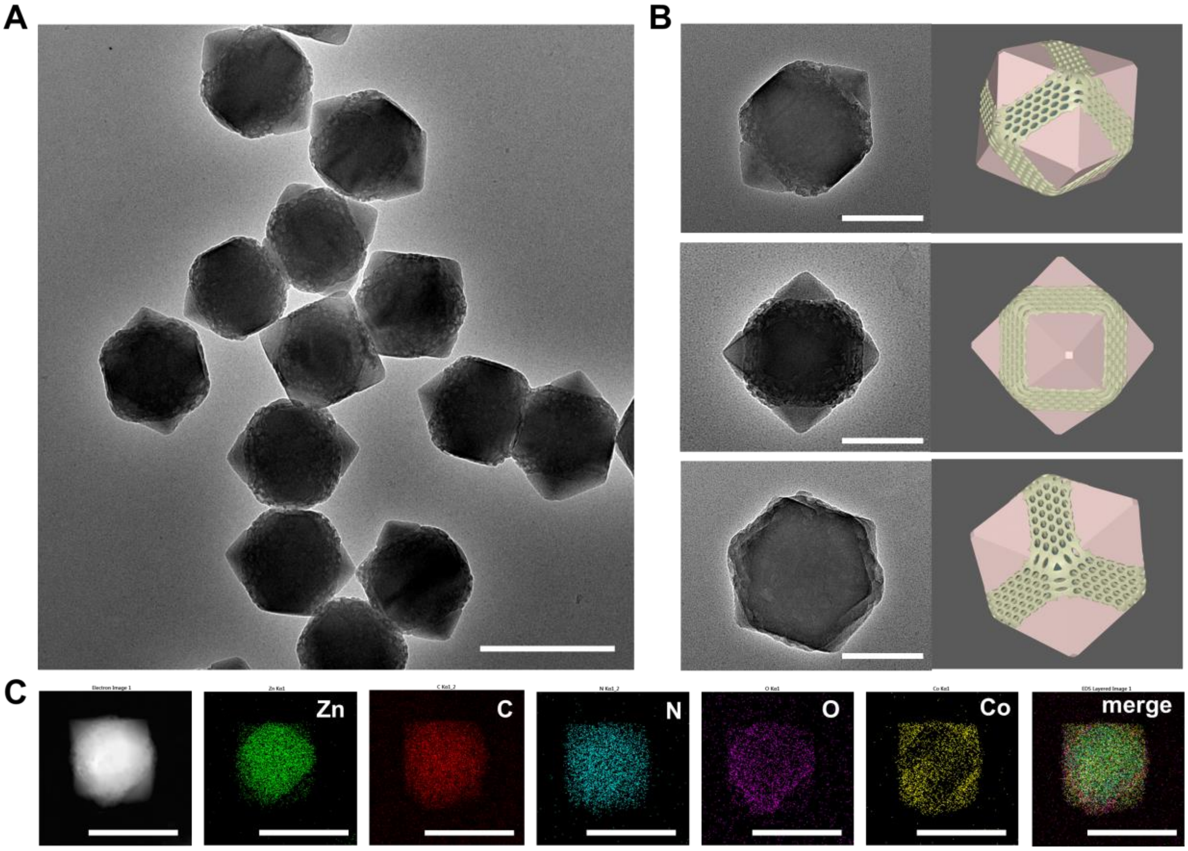

Supplementary Figure 20. (A) TEM image of type 4 p-ANHs. (B) TEM images and corresponding models of a single type 4 p-ANHs taken from different perspectives. (C) EDX elemental mapping of one typical type 4 p-AHN. Scale bars: 500 nm for (A), 200 nm for (B), 250 nm for (C). Similar with type 2 p-ANHs, the type 4 p-AHN are uniform and well dispersed, and the mesopores can be clearly observed in the nanoparticles. The distribution of Co element is consistent with the structural model.

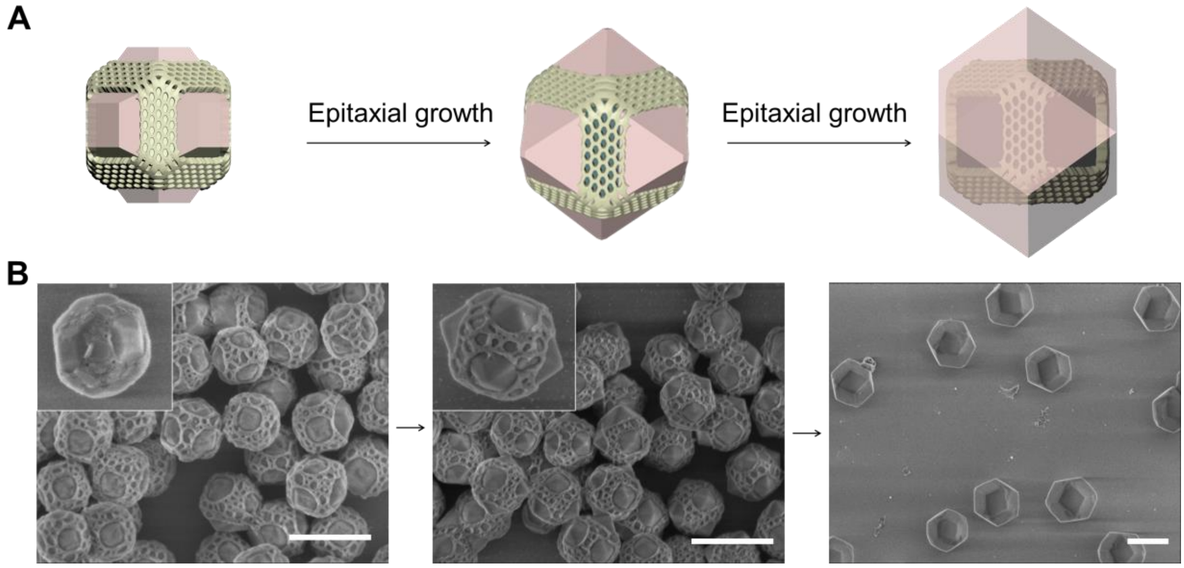

Supplementary Figure 21. (A) The schematic illustration of the epitaxial growth of ZIF-67 on the type 2 p-ANHs. (B) The corresponding SEM images of the products obtained at different stages. Due to the inhibition effect of mPDA on {110} facets, the morphology of the newly formed ZIF-67 building blocks on the exposed {100} facets gradually grow from quadrangular-frustum pyramid to rectangular pyramid. Finally, the regular rhombic dodecahedron nanoparticles are formed, which are composed by ternary building blocks of pristine ZIF-8, mPDA cubical skeleton inlay, outermost ZIF-67. Scale bars: 400 nm.

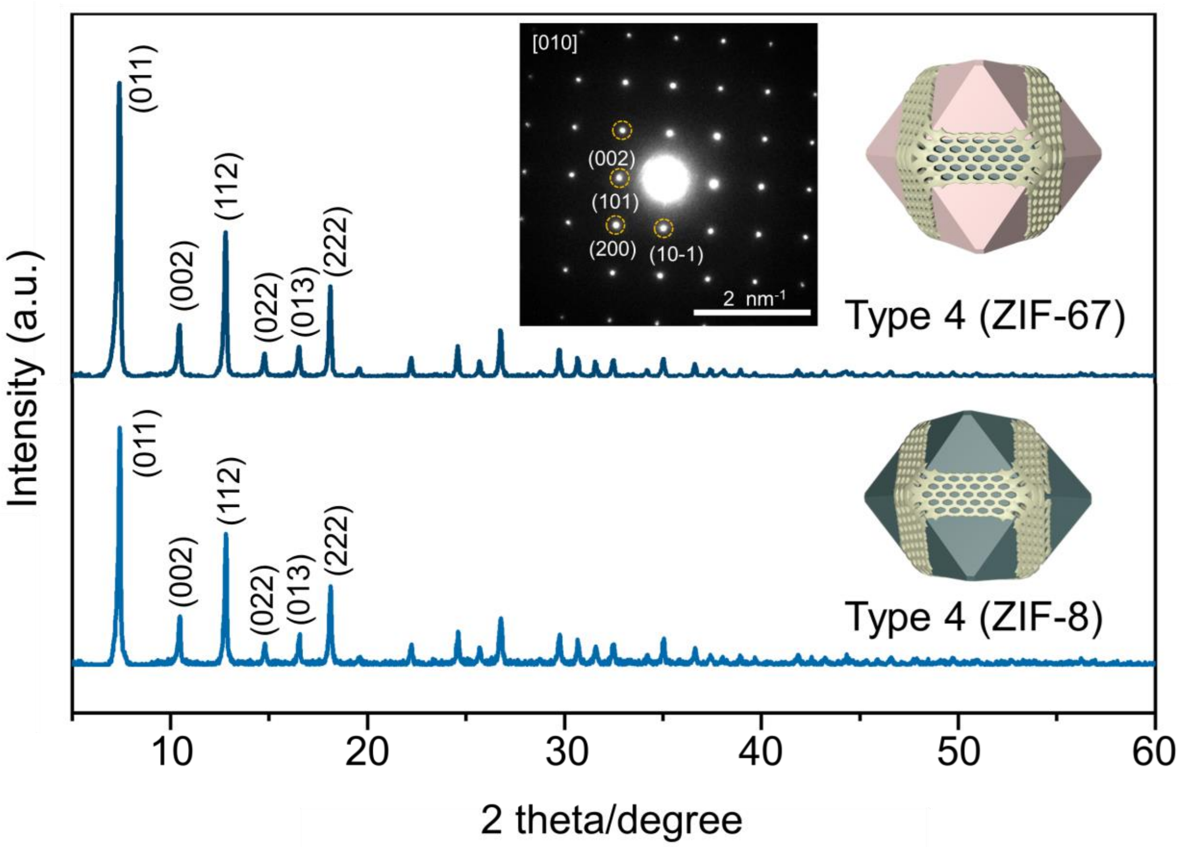

Supplementary Figure 22. XRD patterns of the type 4 p-ANHs after the secondary epitaxial growth of ZIF-8 or ZIF-67 on the {100} facets of type 2 p-ANHs. Because of the identical crystal structure of ZIF-8 and ZIF-67, type 4 p-ANHs with the ternary building block of ZIF-8 and ZIF-67 exhibit the similar diffraction peaks. The inset in A is the SAED patterns taken from an entire type 4 p-AHN along [010] zone axis, indicating the single single-crystalline nature of the obtained type 4 p-ANHs and the epitaxial growth manner of the tertiary building blocks. Source data are provided as a Source Data file.

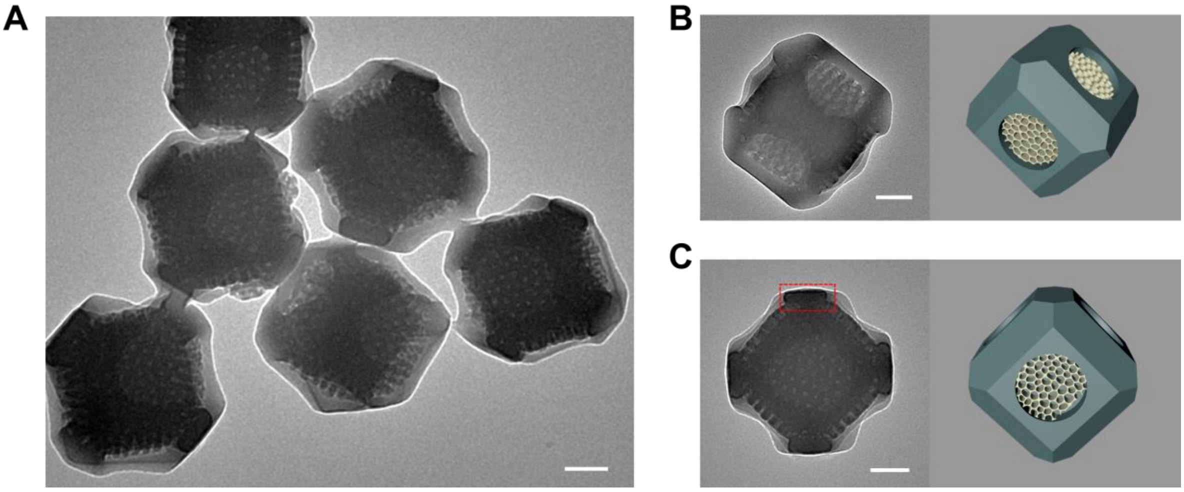

Supplementary Figure 23. (A) TEM image of type 3 p-ANHs with the ternary building block of ZIF-8. (B, C) TEM images and the corresponding diagrams of a single nanoparticle taken from different perspectives. Scale bars: 100 nm for (A-C). It can be seen that the nanohybrids are uniform and well dispersed, and the mesopores can be clearly observed in the nanohybrids. TEM images of a single particle from different perspectives show that the projected contours of the nanoparticles fit well with the corresponding models. As shown in C, there are four high contrast regions at the vertices of the projected square (marked with red line), corresponding to the newly formed tertiary ZIF-8 building blocks by the secondary epitaxial growth. The newly formed tertiary ZIF-8 building blocks are superimposed on the mPDA nanoplates, demonstrating the inlay structure of mPDA in the type 3 architecture.

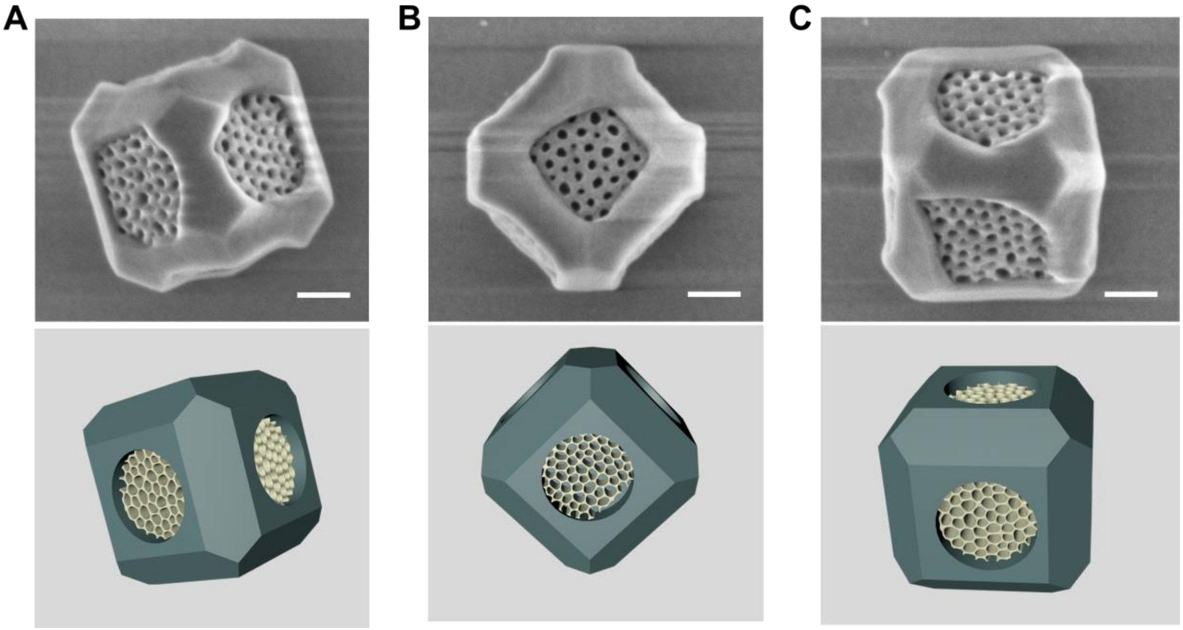

Supplementary Figure 24. (A-C) SEM images and the corresponding diagrams of a single particle of type 3 p-ANHs with the ternary building block of ZIF-8 taken from different perspectives. Scale bars: 100 nm for (A-C). Due to the inhibition effect of mPDA nanoplates on {100} facets, the tertiary MOF building blocks can only epitaxially grow along [110] direction. With the continuous growth of tertiary ZIF-8 building blocks, mPDA nanoplates are partially covered by the tertiary MOF building blocks to form the inlay structure.

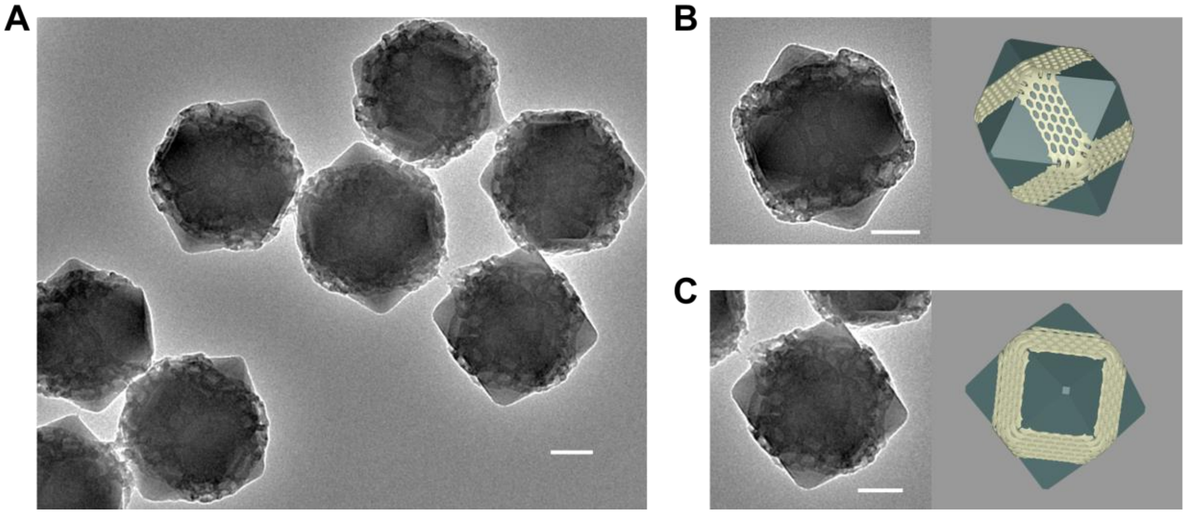

Supplementary Figure 25. (A) TEM image of type 4 p-ANHs with the ternary building block of ZIF-8. (B, C) TEM images and corresponding models of a single nanoparticle taken from different perspectives. Scale bars: 100 nm for (A-C). It can be seen that the nanohybrids are uniform and well dispersed. The mesopores can be clearly observed in the nanoparticles. TEM images of a single particle taken from different perspectives show that the projected contours of the nanoparticles fit well with the corresponding models.

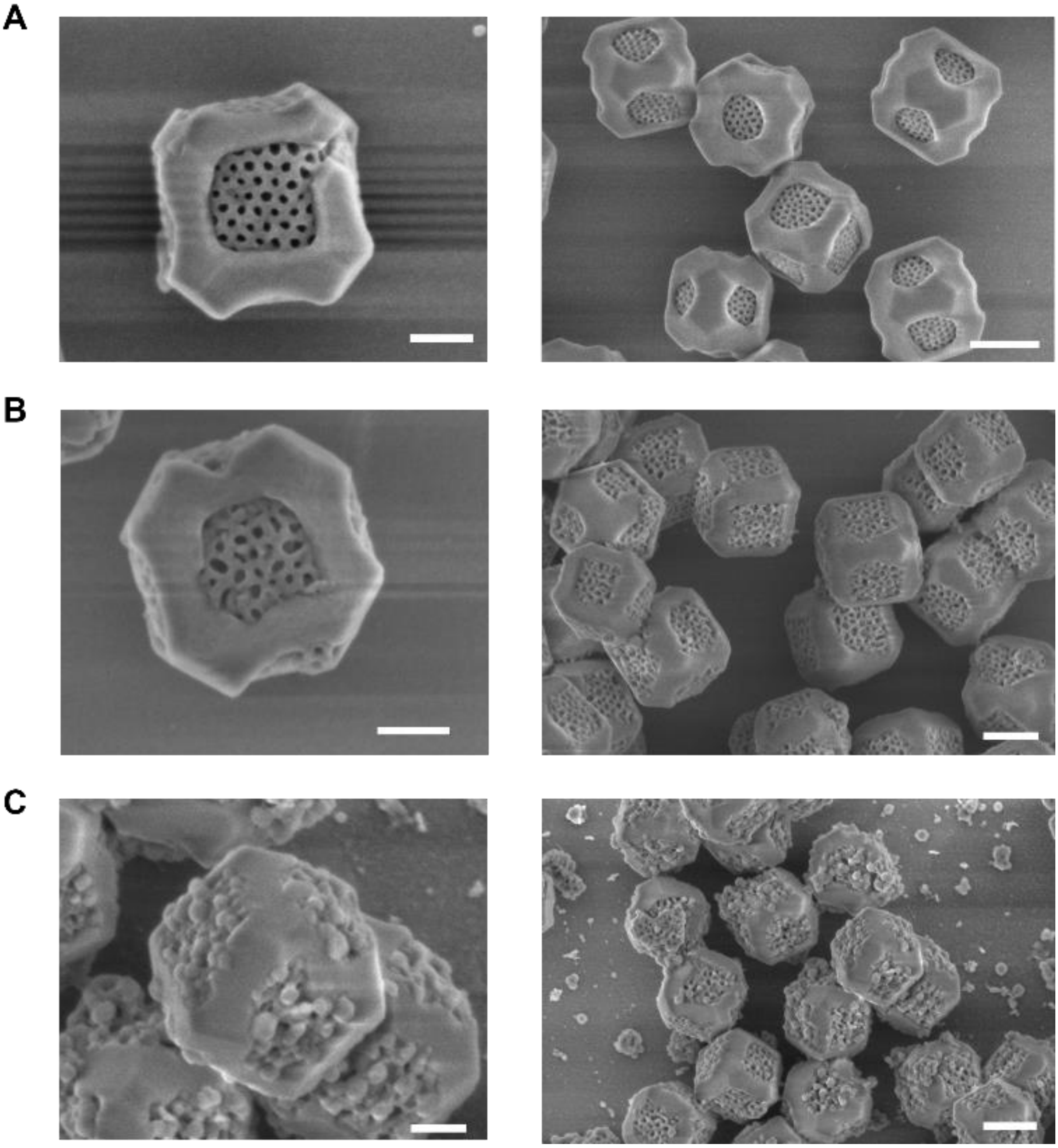

Supplementary Figure 26. TEM images of type 3 p-ANHs synthesized by using different amount of $Co(NO_3)_2$ solution and 2-MeIM solution. (A) 3.0 mL of 2-MeIM solution (25 mM) and 3.0 mL of $Co(NO_3)_2$ solution (12.5 mM); (B) 5.0 mL of 2-MeIM solution (25 mM) and 5.0 mL of $Co(NO_3)_2$ solution (12.5 mM); (C) 7.0 mL of 2-MeIM solution (25 mM) and 7.0 mL of $Co(NO_3)_2$ solution. Scale bars: 100 nm.

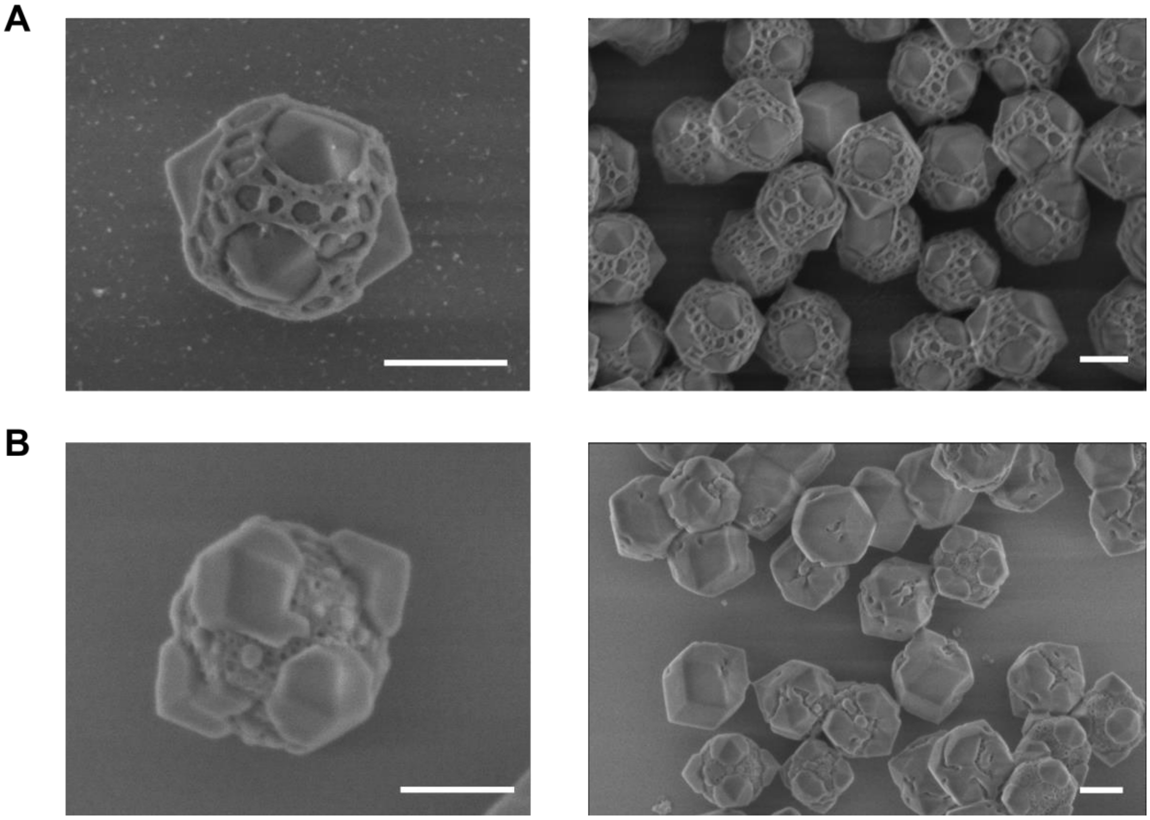

Supplementary Figure 27. TEM images of type 4 p-ANHs synthesized by using different amount of $Co(NO_3)_2$ solution and 2-MeIM solution. (A) 3.0 mL of 2-MeIM solution (25 mM) and 3.0 mL of $Co(NO_3)_2$ solution (12.5 mM); (B) 5.0 mL of 2-MeIM solution (25 mM) and 5.0 mL of $Co(NO_3)_2$ solution (12.5 mM). Scale bars: 200 nm.

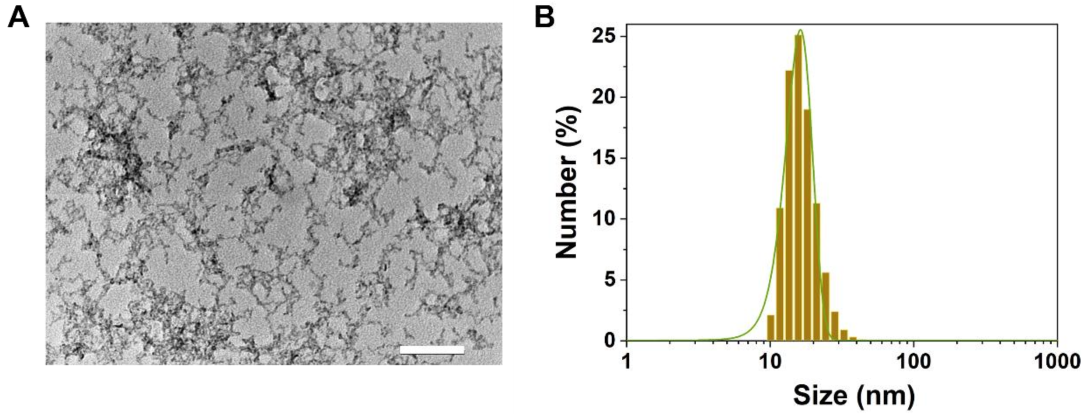

Supplementary Figure 28. (A) TEM image and (B) dynamic light scattering (DLS) measurement of PVP-coated CuO_2 nanodots. It can be seen that the obtained CuO_2 nanodots have a small size of ~7 nm. The CuO_2 nanodots could be well-dispersed in water with a mean hydrodynamic diameter of ~17.3 nm. Scale bars: 100 nm. Source data are provided as a Source Data file.

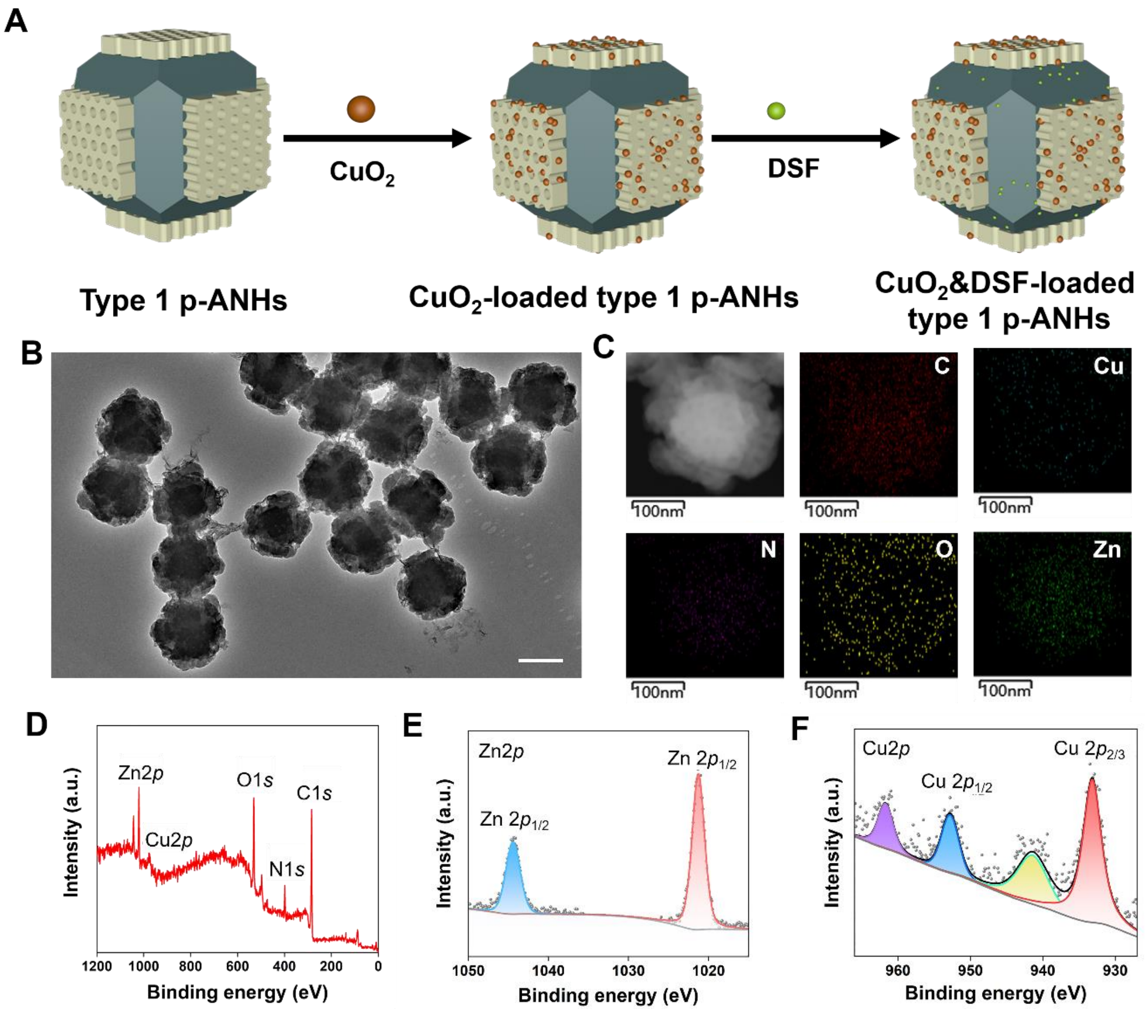

Supplementary Figure 29. (A) Schematic illustration of the synthetic process of CuO_2&DSF-loaded type 1 p-ANHs. (B) TEM image, (C) dark-field TEM image and corresponding elemental mappings of CuO_2&DSF-loaded type 1 p-ANHs. (D−F) XPS spectra of CuO_2&DSF-loaded type 1 p-ANHs in different binding-energy ranges. Scale bars: 200 nm. Source data are provided as a Source Data file. Source data are provided as a Source Data file.

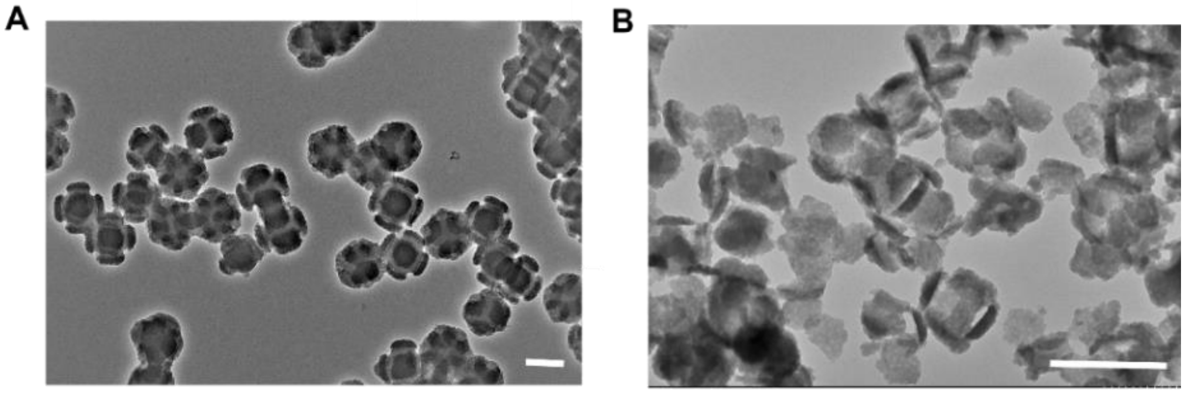

Supplementary Figure 30. TEM images of type 1 p-ANHs after 12 h storage in PBS buffers at (A) pH 7.4 and (B) 6.0. Scale bars: 200 nm. It can be seen that the morphology and structure of type 1 p-ANHs remain intact under normal physiological conditions of pH 7.4, while type 1 p-ANHs decomposes to lamellar dopamine nanosheets under weak acidic condition. These results indicate that type 1 p-ANHs has good pH responsiveness.

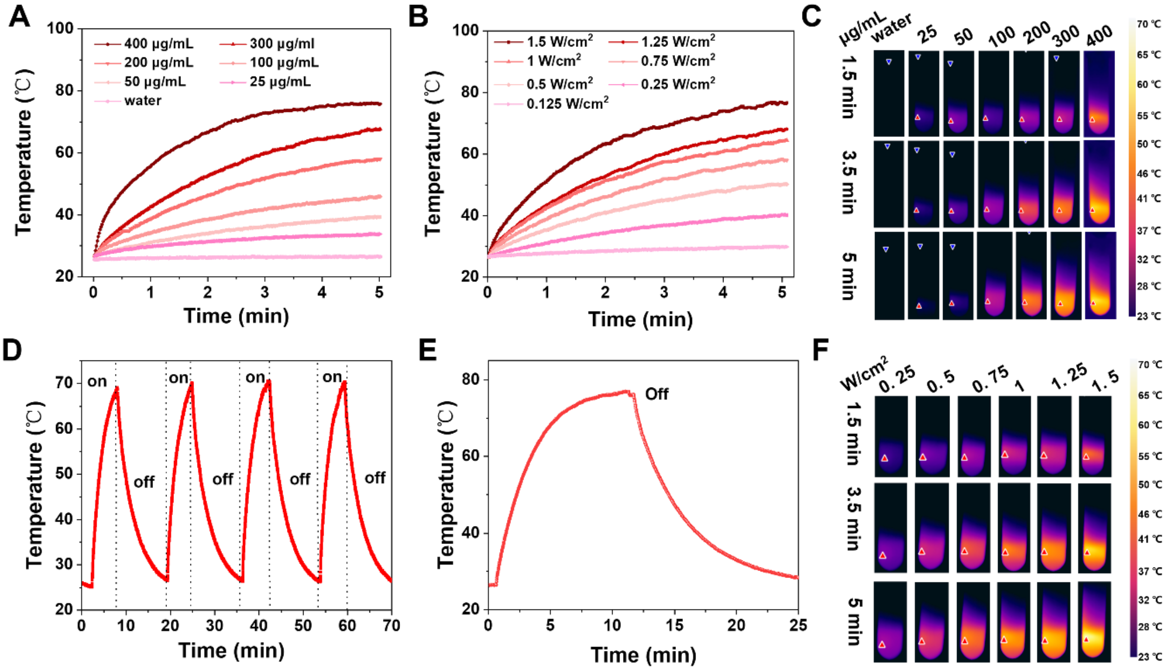

Supplementary Figure 31. (A) Heating curves of CuO_2&DSF-loaded type 1 p-ANHs with different concentrations upon 808-nm laser irradiation (1.25 W/cm^2). (B) Heating curves of CuO_2&DSF-loaded type 1 p-ANHs aqueous dispersion (300 μg/mL) upon 808-nm laser irradiation at varying power densities. (C) Infrared (IR) thermographic images of CuO_2&DSF-loaded type 1 p-ANHs dispersions with different concentrations under 808-nm laser irradiation (1.25 W/cm^2). (D) Heating curve of a CuO_2&DSF-loaded type 1 p-ANHs aqueous dispersion (300 μg/mL) under four cycles of heating and cooling processes (1.25 W/cm^2). (E) Photo-to-heat conversion capability of a CuO_2&DSF-loaded type 1 p-ANHs aqueous dispersion (300 μg/mL) upon 808-nm laser irradiation (1.25 W/cm^2). (F) IR thermographic images of CuO_2&DSF-loaded type 1 p-ANHs solution (300 μg/mL) under the irradiation of 808-nm laser with varied power densities. Source data are provided as a Source Data file.

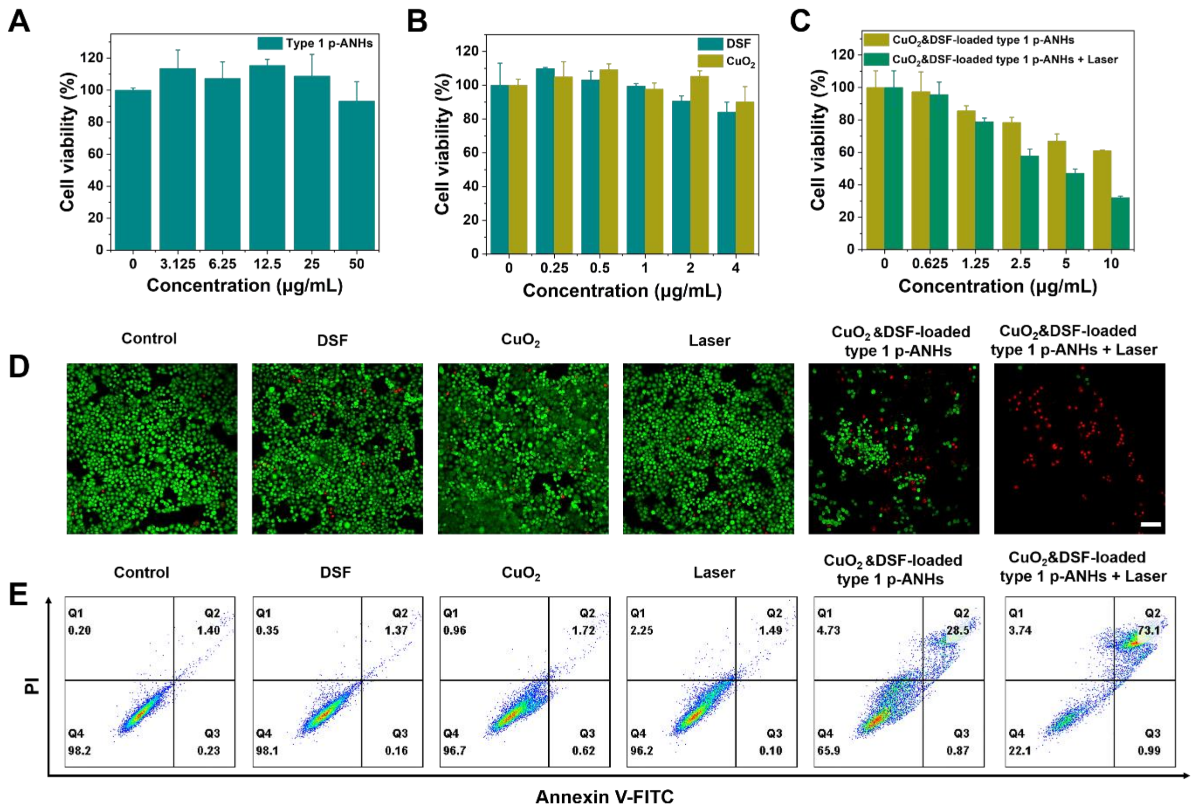

Supplementary Figure 32. (A−C) Viability of HeLa cells after different treatments for 24 h. Error bars represent standard deviation (n = 3 independent experiments). (D) CLSM images of HeLa cells after incubation with PBS, free DSF, CuO_2, Laser, CuO_2&DSF-loaded type 1 p-ANHs, and CuO_2&DSF-loaded type 1 p-ANHs + Laser for 24 h. (E) Quantitative analysis of HeLa cells by flow cytometry, including the percentages of live, early apoptotic, and late apoptotic cells after different treatments. Scale bars: 100 μm. It can be seen that type1 p-ANHs, CuO_2 and DSF alone show negligible toxicity to HeLa cells, even at high concentrations. In comparison, more than 40% of cells were killed after the CuO_2&DSF-loaded type 1 p-ANHs treatment (10 μg/mL). Furthermore, the chemotherapeutic efficacy of CuO_2&DSF-loaded type 1 p-ANHs was significantly increased when the cells were further treated with NIR laser. Source data are provided as a Source Data file.

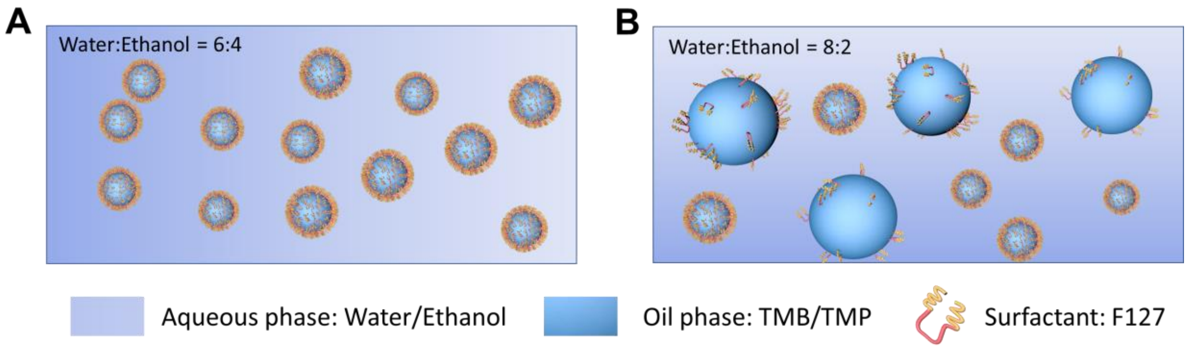

Supplementary Figure 33. The schematic illustration of the emulsion system formed by the water/ethanol, TMB/TMP and F127 surfactants: (A) water/ethanol ratio of 6:4, (B) water/ethanol ratio of 8:2. When the water/ethanol ratio is 6:4, TMB/TMP oil phase can form stable nanodroplets. In this case, the oil nanodroplets are well encapsulated in the surfactant micelles. In contrast, the increase of water/ethanol ratio to 8:2 can induce the decrease of emulsion stability, resulting in phase separation and the formation of large metastable oil droplets. The oil droplets are unstable and have wide and variable size distributions.

Supplementary Tables

Supplementary Table 1.

The porosity analysis of the obtained p-ANHs with different architectures.

Architecture	S_{total} (m^2/g)	V_{total} (cm^3/g)	S_{micro} (m^2/g)	V_{micro} (cm^3/g)	S_{meso} (m^2/g)	V_{meso} (cm^3/g)	mPDA mass ratio (%)
Pristine ZIF-8	1493	0.81	1457	0.74	25	0.068	0
Type 1	672	0.53	569	0.29	84	0.23	47.6
Type 2	618	0.67	508	0.26	93	0.33	49.1
Type 3 (ZIF-8)	1092	0.52	1074	0.48	13	0.068	7.69
Type 3 (ZIF-67)	1199	0.57	1162	0.52	24	0.085	7.60
Type 4 (ZIF-8)	1218	0.56	1195	0.53	14	0.058	8.98
Type 4 (ZIF-67)	1043	0.52	1008	0.46	25	0.10	8.95

Supplementary Table 2.

The adsorption energy of DHI-dimer and TMP on {100} and {110} facets with the presence of implicit solvent model. All the configuration are firstly optimized in vacuum, followed by single point energy calculations with different value of permittivity setup. (unit: eV)

	DHI/{100}	TMP/{100}	DHI/{110}	TMP/{110}
No solvent	1.21	1.27	1.15	0.44
ε=78.4	1.77	1.99	0.69	0.18
ε=60.0	1.76	1.97	0.72	0.20
ε=40.0	1.74	1.94	0.76	0.22

Supplementary References

1. Liu, C. et al. Site-specific growth of MOF-on-MOF heterostructures with controllable nanoarchitectures: beyond the combination of MOF analogues. Chem. Sci. **11,** 3680-3686 (2020).

2. Lin, L. S. et al. Synthesis of copper peroxide nanodots for H_2O_2 self-supplying chemodynamic therapy. J. Am. Chem. Soc. **141,** 9937-9945 (2019).
